# The Increase in Phosphorylation Levels of Serine Residues of Protein HSP70 during Holding Time at 17°C Is Concomitant with a Higher Cryotolerance of Boar Spermatozoa

**DOI:** 10.1371/journal.pone.0090887

**Published:** 2014-03-06

**Authors:** Marc Yeste, Efrén Estrada, Maria-Montserat Rivera del Álamo, Sergi Bonet, Teresa Rigau, Joan-Enric Rodríguez-Gil

**Affiliations:** 1 Unit of Animal Reproduction, Department of Animal Medicine and Surgery, Faculty of Veterinary Medicine, Autonomous University of Barcelona, Barcelona, Spain; 2 Biotechnology of Animal and Human Reproduction (TechnoSperm), Department of Biology, Institute of Food and Agricultural Technology, University of Girona, Girona, Spain; Cornell University College of Veterinary Medicine, United States of America

## Abstract

Boar-sperm cryopreservation is not usually performed immediately after semen collection, but rather a holding time (HT) of 4 h–30 h at 17°C is spent before starting this procedure. Taking this into account, the aim of this study was to go further in-depth into the mechanisms underlying the improving effects of HT at 17°C on boar-sperm cryotolerance by evaluating the effects of two different HTs (3 h and 24 h) on overall boar-sperm function and survival before and after cryopreservation. Given that phospho/dephosphorylation mechanisms are of utmost importance in the overall regulation of sperm function, the phosphorylation levels of serine residues (pSer) in 30 different sperm proteins after a 3 h- or 24 h-HT period were also assessed. We found that a HT of 24 h contributed to a higher sperm resistance to freeze-thawing procedures, whereas mini-array protein analyses showed that a HT of 24 h induced a significant (*P*<0.05) increase in pSer (from 100.0±1.8 arbitrary units in HT 3 h to 150.2±5.1 arbitrary units in HT 24 h) of HSP70 and, to a lesser extent, in protein kinases GSK3 and total TRK and in the cell-cycle regulatory protein CDC2/CDK1. In the case of HSP70, this increase was confirmed through immunoprecipation analyses. Principal component and multiple regression analyses indicated that a component explaining a percentage of variance higher than 50% in sperm cryotolerance was significantly correlated with pSer levels in HSP70. In addition, from all the parameters evaluated before freeze-thawing, only pSer levels in HSP70 resulted to be able to predict sperm cryotolerance. In conclusion, our results suggest that boar spermatozoa modulate its function during HT, at least partially, by changes in pSer levels of proteins like HSP70, and this is related to a higher cryotolerance.

## Introduction

Mammalian sperm cryopreservation is a stressful event that generates damaged spermatozoa through mechanisms such as oxidative stresses and cold-shock (See Rath et al. [1], for a review). In the case of porcine spermatozoa, cryopreservation is usually performed after sperm has been stored for up to a 24-h period (Holding Time, HT) at 17°C after collection, as this period in contact with seminal plasma has been reported to yield higher tolerance to low temperatures [2]–[4]. However, the information about the effects of HT on post-thaw boar-sperm function and survival has been scarce and inconsistent so far. Thus, while Kotzias-Bandeira et al. [5] and Eriksson et al. [6] found that a longer HT, rather than a shorter one, was beneficial for post-thaw sperm viability, Guthrie and Welch [7] found no significant HT effect on post-thaw sperm survival. These discrepancies can be related to the alternate sperm processing procedures utilised by each investigation. In any case, more information is needed to reach a consensus on this point.

Mature spermatozoa are transcriptionally quiescent cells that are not able to regulate gene expression. This implies that sperm cannot modulate gene expression to face stressful environmental conditions and to modulate its physiology. Related to this, post-translation protein modifications like phospho/dephosphorylation are known to play a significant role in some mechanisms regulating sperm function and response to environmental stress [8]–[11]. In fact, protein phospho/dephosphorylation is a general mechanism present in all cells that plays a major role in a wide array of cellular processes [12]. As indicated above, in the case of mature mammalian spermatozoa, protein phospho/dephosphorylation is of the utmost importance as it is involved in the regulation of processes such as the control of sperm motility [10], [13], sperm capacitation [14]–[16], response to osmotic stress [17], zona pellucida recognition and acrosome reaction [18]–[19].

To the best of our knowledge, no previous studies have hitherto investigated whether the differences between shorter and longer HTs are related to changes in phosphorylation patterns of some relevant sperm proteins. For this reason, in this present study we studied the effect of HT on the resistance to cryopreservation of boar spermatozoa. We investigated how two different HTs (3 h and 24 h) affect nucleoprotein structure, DNA fragmentation, sperm membrane integrity and lipid disorder, and sperm motility, amongst other sperm parameters, before (Ext, extended semen) and after freeze-thawing (FT, frozen-thawed spermatozoa). In addition, an aliquot of these ejaculates was taken after a HT of 3 h and of 24 h and immediately before starting sperm cryopreservation to compare the phosphorylation levels of serine residues (pSer) in a set of 30 sperm proteins that may be involved in the modulation of sperm function and that have been studied in a previous report by our group [11]. Within these proteins, there are cell-cycle controlling proteins like cyclins [20]; stress-modulating proteins, like heat-shock protein 70 (HSP70, also known as HSPA1A), and others related to apoptosis like caspase 9 [21]–[24], and cell-cell adhesion proteins, such as clusterin [25]–[26]. Additionally, we have also studied specific protein kinases, like PKA and PKC [14], [27]–[28], and phosphatases, such as PP1, PP2A, PP2B, PTP1 and PTP2, as both are involved in the regulation of sperm function [13], [15]–[16], [19], [29]. Finally, other protein kinases that also play important roles in the control of capacitation and resistance to oxidative stress in mammalian spermatozoa, such as glycogen synthase kinase-3 (GSK3, see [10]), ERK-1 and ERK-2 [30]–[31] and the AKT-phosphoinositide 3-kinase (PI3K) system [32]–[33], have also been included in our study. Given that the results obtained from mini-array analyses, immunoprecipitation-confirming studies were performed on a single protein, HSP70, in order to further clarify the relationship between HT, cryotolerance and specific pSer changes of this protein in boar spermatozoa.

## Materials and Methods

### Sperm Samples

In this study, boars were not handling by us, the semen was obtained from a local farm (the farm - Servicios Genéticos Porcinos, S.L.; Roda de Ter, Barcelona, Spain). Thus, such ejaculates were initially dedicated for artificial insemination purposes, and we just bought them for our experimental purposes. Despite all the aforementioned, and even though it was not required as the authors did not manipulate any boar, the experimental protocol was approved by the Ethics Committee of our institution. This ethics committee was known as “Bioethics Commission of the Autonomous University of Barcelona” (Bellaterra, Cerdanyola del Vallès, Spain).

Overall, twelve ejaculates coming from twelve healthy and mature boars of Pietrain pure breed were used (age: 21.5±0.9 months; means±standard error of the mean, SEM). Animals were housed in climate-controlled buildings, fed with an adjusted diet (2.3 Kg·day^−1^) consisting of a basal diet and a 1% premix for boars (P174N; TecnoVit; Tarragona, Spain), and provided with water *ad libitum*. Ejaculates were collected twice per week by the gloved-hand technique with an interval of at least three days between collections. After removing the gelatinous fraction by filtering through gauze, the total volume of the sperm-rich fraction was diluted 1∶1 (v:v) in a short-term Beltsville Thawing Solution (BTS)-based extender (Cidosa, TecnoVit; Tarragona, Spain). The diluted sperm-rich fractions were cooled to 17°C by placing them in a 17°C mobile-incubator and immediately transported at this temperature in an insulated container to our laboratory within two hours.

### Experimental Design

Upon arrival, the quality of the ejaculates was evaluated to check that they satisfied the quality standard (total sperm motility>80%, morphologically normal spermatozoa and sperm viability>85%; See [34]). Since the quality of these twelve ejaculates was over the set thresholds, they were all included in our study. Next, each ejaculate was split into two fractions of equal volume. One of these fractions was frozen 3 h after collection (HT = 3 h), while the other was stored at 17°C up to 24 h after collection (HT = 24 h). Before starting the cryopreservation process, two aliquots of 10 mL each were taken (i.e. after either 3 h or 24 h of semen collection), one of each for evaluating sperm parameters (Ext, Extended semen) and the other for performing the protein assessment (mini-arrays and immunoprecipation studies). The protein-assessment aliquot was centrifuged at 600×*g* and at 17°C for 5 min, the supernatant was discarded and the pellet was frozen in liquid nitrogen. The remaining volume of both fractions (i.e. HT = 3 h and HT = 24 h) was cryopreserved at each relevant time point, following the procedure described in Section 2.3, and stored in liquid nitrogen for at least two months. After thawing accordingly the procedure described in Section 2.3, samples were diluted with three volumes of warmed (37°C) BTS ([35]; final dilution: 1/4) and incubated at 37°C for 30 min and 240 min (FT, Frozen-thawed spermatozoa), prior to determining sperm functional parameters (through motility and flow cytometry assessments), DNA fragmentation and free cysteine radicals in sperm nucleoproteins, as an indication of disrupted disulphide bonds. Two time-points (30 min and 240 min) were chosen to evaluate the sperm cells after freeze-thawing, the last one being set to assure the survival of FT spermatozoa within the insemination-to-ovulation interval recommended for cryopreserved doses [36] and as a sperm resistance test.

### Cryopreservation and Thawing of Sperm Samples

Semen samples were cryopreserved using the Westendorf method [37], as modified by Yeste et al. [34]. All of the ejaculates were centrifuged at 17°C and at 600×*g* for 5 min. Pellets were then recovered with 3 mL-4 mL of the remaining supernatant and diluted to a concentration of 1.5×10^9^ spermatozoa·mL^−1^ (using a Makler counting chamber; Sefi-Medical Instruments; Haifa, Israel) in a freezing medium (LEY) containing lactose (80%, v:v; 310 mM) and egg yolk (20%, v:v). Next, spermatozoa were cooled down to 5°C for 120 min (cooling ramp: 0.1°C·min^−1^) using a programmable freezer (Icecube14S-B; Minitub Ibérica, SL; Tarragona, Spain) and subsequently diluted at 1×10^9^ spermatozoa·mL^−1^ in LEYGO extender that contained 6% glycerol (Sigma) and 1.5% Orvus ES Paste (OEP, Equex STM; Nova Chemical Sales Inc.; Scituate; MA, USA). Final concentrations of glycerol and OEP in cryopreserved samples were 2% and 0.5%, respectively. Spermatozoa were finally packed in 0.5-mL plastic straws (Minitub Ibérica, SL) and transferred to a programmable freezer (Icecube14S-B; Minitub Ibérica SL). The freezing programme (SY-LAB software; Minitub Ibérica SL) consisted of 313 sec of cooling at the following rates: −6°C·min^−1^ from 5°C to −5°C (100 sec), −39.82°C·min^−1^ from −5°C to −80°C (113 sec), maintained for 30 sec at −80°C, and finally cooled at −60°C·min^−1^ from −80°C to −150°C (70 sec). The straws were then plunged into liquid N_2_ (−196°C) for further storage.

After being stored in liquid N_2_ for at least two months only for schedule reasons, samples were thawed and evaluated. With this purpose, four straws per ejaculate were thawed and diluted with three volumes of warmed BTS at 37°C (at a final dilution of 1/4). Each straw was shaken individually for 20 sec in a water bath at 37°C.

### Flow Cytometric Analyses

Information about flow cytometry analyses is given according to the recommendations of the International Society for Advancement of Cytometry (ISAC) [38]. These analyses were conducted to evaluate some functional parameters of spermatozoa, such as plasma membrane integrity and permeability, membrane lipid disorder, intracellular calcium levels, or ROS levels in extended and FT spermatozoa after a HT of 3 h or 24 h. In each case, sperm concentration was adjusted to 1×10^6^ spermatozoa·mL^−1^ in a final volume of 0.5 mL, and spermatozoa were then stained with the appropriate combinations of fluorochromes. Plasma membrane integrity was assessed through SYBR-14/PI assay according to the protocol described by Garner and Johnson [39], as well as through PNA-FITC/PI co-staining following the procedure described by Nagy et al. [40]. In addition, changes in the permeability of sperm plasma membrane were evaluated through co-staining with YO-PRO-1 and PI, following Martin et al. [24], and membrane lipid disorder was assessed using the protocol for Merocyanine 540 (M-540) and YO-PRO-1 described by Harrison et al. [41]. Intracellular calcium levels of spermatozoa were determined through Fluo3-AM/PI co-staining [42]. Levels of peroxides and superoxides were evaluated through H_2_DCFDA/PI and HE/YO-PRO-1, respectively, according the protocol described by Guthrie and Welch [43]. Finally, data was corrected following Petrunkina et al. [44] by determining the percentage of non-DNA-containing particles, to avoid an overestimation of sperm particles. All protocols are described in detail in Information S1.

In all cases, samples were evaluated through a Cell Laboratory QuantaSC™ cytometer (Beckman Coulter; Fullerton, CA, USA; Serial Number: AL300087) using single-line visible light (488 nm) from an argon laser. A minimum of 10,000 events per replicate was evaluated, and data was collected in List-mode Data files (.LMD) and analysed using the Cell Lab Quanta SC MPL Analysis Software (version 1.0; Beckman Coulter). In all cases except for the SYBR-14/PI assessment, data obtained from flow cytometry experiments were corrected according to the procedure set by Petrunkina et al. [44]. Each assessment for each sample and parameter was repeated three times in independent tubes, prior to calculating the corresponding mean±SEM. Technical details are also given in Information S1.

### Determination of Free Cysteine Radicals in Sperm Nucleoproteins

The determination of free cysteine radicals in sperm nucleoproteins, as a measure of disrupted disulphide bonds, was carried out before (extended) and after freeze-thawing (FT) by following the protocol adapted to boar spermatozoa by Flores et al. [45] (See Information S1 for details). The results obtained after reading at 343 nm were normalised through a parallel determination of the total protein content of samples, using a commercial kit (Quick Start™ Bradford Protein Assay; BioRad, Hercules; CA, USA) for the Bradford method [46].

### DNA Fragmentation Analysis

Sperm DNA fragmentation was assessed before (extended) and after freeze-thawing (FT) using a sperm chromatin dispersion test (SCDt) specifically designed for boar spermatozoa (Sperm–Halomax–Sus for fluorescence microscopy; ChromaCell S.L.; Madrid, Spain). This test is based on the different response that intact and fragmented DNA show after a de-proteinisation treatment, and previous reports have shown that the results obtained with this technique strongly correlate with those obtained with other tests, like the neutral comet assay [47], [48]. Protocol details are provided with a great detail in Information S1. Samples were observed under an epifluorescence microscope (Zeiss AxioImager Z1; Karl Zeiss) at 40× magnification and three counts of 250 spermatozoa each were made per sample. Spermatozoa with fragmented DNA presented a large and spotty halo of chromatin dispersion, while spermatozoa with non-fragmented DNA showed a small halo.

### Analysis of Sperm Motility

Sperm motility analysis was performed by utilising a commercial computer assisted sperm analysis (CASA) system (Integrated Sperm Analysis System V1.0; Proiser; Valencia, Spain), 15 µl of each sperm sample (at a concentration of 1–3×10^7^ spermatozoa·mL^−1^) being placed in a Makler counting chamber (Sefi-Medical Instruments). Our CASA system was based upon the analysis of 25 consecutive digitalised photographic images obtained from a single field at a magnification of 10× in a negative phase-contrast field. These 25 consecutive photographs were taken in a time lapse of 1 sec, which implied a velocity of image capturing of one photograph every 40 msec. Five to six separate fields were taken for each replicate, and three replicates were run per sample. The sperm motility descriptors obtained were those described by Yeste et al. [49], and the settings taken into account for all of the utilised motility parameters are provided as Information S1. Total motility (%TMOT) was defined as the percentage of spermatozoa that showed a VAP>10 µm·s^−1^, whereas progressive motility (%PMOT) was defined as the percentage of spermatozoa that showed a VAP>45 µm·s^−1^.

### Mini-array Analysis of Serine Phosphorylation of 30 Selected Proteins

**Figure 1:**
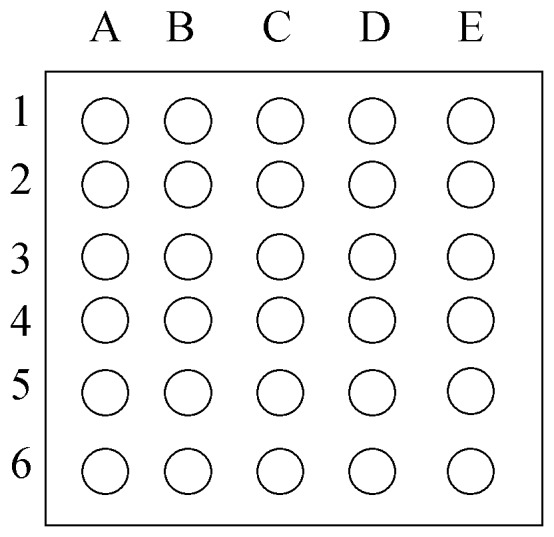
General scheme of the distribution of the proteins for the mini-array analysis. 1A: AKT-1/AKT-2. 1B: CDK6. 1C: CYCLIN E. 1D: IRAK. 1E: PYK2/CAKb. 2A: CASPASE 9. 2B: C-KIT. 2C: CYCLIN H. 2D: PI3 KINASE/p85. 2E: C-RAF-1. 3A: CDC25. 3B: ERK-1. 3C: PKC. 3D: RAS. 3E: CDC6. 4A: CLUSTERIN. 4B: ERK-2. 4C: PP1, PP2A, PP2B, PPX. 4D: TRK A, B, C. 4E: CDK1/CDC2. 5A. CYCLIN A. 5B: GSK-3a. 5C: PTP1 (SH). 5D: CDK2. 5E: CYCLIN B. 6A: HSP70. 6B: PTP1 B. 6C: CDK4. 6D: CYCLIN D3. 6E: PTP2 (SH).

The phosphorylation levels of serine residues in 30 sperm proteins (Figure 1) in semen samples being at two different HTs (i.e. 3 h and 24 h) were assessed using customised mini-arrays provided by Hypromatrix Inc. (Worcester, MA, USA). With this aim, twenty-four aliquots coming from twelve different ejaculates (stored after a HT of 3 h or of 24 h) were used and processed as described in Information S1 As stated in Introduction, these proteins were chosen following previous results obtained from our laboratory and published in Fernández-Novell et al. [11].

The intensity of the spots was quantified using specific software for image analysis of blots and arrays (Multi Gauge v3.0; Fujifilm Europe; Düsseldorf, Germany), in which the background was previously made uniform for all of the arrays analysed. The values obtained for the HT of 3 h were transformed in order to obtain a basal arbitrary value of 100, from which the intensity values for the other samples (i.e. HT 24 h) were calculated. For each sperm sample (stored for 3 h or 24 h), two replicates were assessed prior to calculating the corresponding mean±SEM. Furthermore, two types of negative control were applied. In one, three arrays were incubated with a randomly chosen sample but without further incubation with the antibody. In the other, three arrays were incubated with the antibodies but without samples.

### Immunoprecipitation against HSP70 and Western-blot Assessments

Results obtained in mini-array analyses were confirmed through immunoprecipitation analyses (immunoprecipitation kit code product 17-6002-35; Healthcare Bio-Sciences AB; Uppsala, Sweden) against human HSP70, using a monoclonal antibody commercially available (ADI-SPA-810, Enzo Life Sciences; New York; NY, USA). With this purpose, twenty-four aliquots (5 mL each) from the same twelve sperm samples used in the other assessments and stored both for 3 h (HT = 3 h) or 24 h (HT = 24 h), were taken at each relevant time point and centrifuged at 600×g and 17°C. With this method, described with a great detail in Information S1, we isolated HSP70 from sperm protein extracts. Then, samples were evaluated through Western Blot assessment with anti-HSP70 and anti-phosphorylated serines (HM2070, Hypromatrix Inc.; Worcester, MA, USA) antibodies. This allowed us to calculate, per sample, a ratio between the mark intensities of phosphorylated serines and HSP70 (pSer:total HSP70). Two replicates for each sperm sample/HT were evaluated prior to calculating the corresponding mean±SEM per ratio/sperm sample.

### Statistical Analyses

Statistical analyses were conducted using IBM SPSS 19.0 (IBM corp.; Chicago, Illinois) and SYSTAT 12.0 for Windows statistical packages (SYSTAT Software Inc.; Evanston, IL, USA), and data are presented as mean±SEM. Each sperm sample held at a given HT was considered as an independent observation, and the minimal level of significance was set at *P*<0.05 in all statistical analyses.

Data obtained from the analysis of all sperm parameters, as well as from the assessments of protein mini-arrays and phosphorylated serine residues in immunoprecipitation studies anti-HSP70 were tested for normality and homogeneity of variances using the Shapiro-Wilk and Levene tests. When necessary, data were transformed using the arcsine square root (arcsin √x) to match the parametric assumptions.

### General Linear Models

In the case of sperm parameters (i.e. sperm membrane integrity and permeability, intracellular calcium, peroxide and superoxide levels, free cysteine radicals in sperm nucleoproteins, DNA fragmentation, and sperm motility and velocity descriptors), a generalised linear mixed model for repeated measures was run where each sperm parameter was the dependent variable, incubation time at 37°C (30 min or 240 min) was the intra-subject factor, and the storing method and HT (i.e. Ext 3 h, Ext 24 h, FT 3 h or FT 24 h) and the ejaculate were, respectively, the fixed-effect and random-effect factors. A post hoc Bonferroni’s test was used for pair-wise comparisons.

In the case of phosphorylated serine residues in mini-array protein and in anti-HSP70 immunoprecipitation analyses, differences in spots-intensity or pSer:total HSP70 ratios were compared through a Student’s t-test for related samples, where each protein in the case of mini-arrays or pSer:total HSP70 ratios in the case of immunoprecipitation assessments were the dependent variables, and the HT (3 h or 24 h) was the factor.

### Calculation of Cryotolerance Indexes

For each sperm sample held at a given HT (*a*; i.e. either 3 h or 24 h) and sperm parameter (*x*; i.e. sperm membrane integrity and permeability, intracellular calcium, peroxide and superoxide levels, free cysteine radicals in sperm nucleoproteins, DNA fragmentation, and sperm motility and velocity descriptors), a cryotolerance index (C_x_) was calculated as the quotient between the value of this parameter (*x*) before (i.e. in extended semen, Ext) and after freeze-thawing (FT). The calculation of this index also took into account the time at 37°C (*b*), either 30 or 240 min, at which samples were incubated prior to determining such sperm parameter. The mathematic formula was as follows:
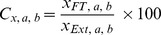



Thus, for example, the cryotolerance index of viable spermatozoa (SYBR-14^+^/PI^−^) evaluated through SYBR-14/PI staining in a given sperm sample held at a HT of 24 h and after both extended and frozen-thawed samples were incubated at 37°C for 240 min was:
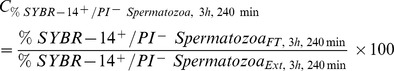



### Principal Component Analyses

After calculating cryotolerance indexes as described in Section 2.10.2, two factorial analyses were run using the values obtained for the crytolerance indexes after 30 and 240 min of incubation at 37°C. In each analysis, these indexes were sorted into some components extracted by principal component analysis (PCA) and the obtained data matrix was rotated using the Varimax procedure with Kaiser normalisation. Only those variables with a square factor loading (*a_ij_^2^*) higher than 0.3 with its respective component, and lower than 0.1 with respect to the other components in the rotated matrix, were selected from the linear combination of *j* variables (*z*) in each component *y_i_* (y_i_ = a_i1_z_1_+ a_i2_z_2_+ …+a_ij_z_j_). Regression factors for each component after PCA were saved and used for multiple regression analyses.

### Correlation and Multiple Regression Analyses

Correlations between all the evaluated sperm parameters were calculated using Pearson correlation. In addition, spots-intensity of pSer levels obtained in mini-array protein and pSer:total HSP70 ratios determined in anti-HSP70 immunoprecipitation analyses were correlated with regression factors from PCA using cryotolerance indexes.

Finally, multiple regression analyses were conducted to determine the ability of all the parameters (*x*) evaluated before freeze-thawing (i.e. all sperm parameters, spots intensity for each protein obtained in mini-array protein analyses, and pSer:total HSP70 ratios from anti-HSP70 immunoprecipitation studies) to predict the cryotolerance of a given ejaculate after a given HT. The procedure used (the forward stepwise model) was the same described by Yeste et al. [50] and consisted of optimising the regression equation to increase the determination coefficient (R^2^). The dependent variable (*y*) in all the cases was the regression factor of the first component that resulted from PCA analyses with cryotolerance indexes, as this component explained the highest percentage of variance in each PCA. The significance level for introducing each parameter in the multiple regression model was 0.10 and the significance level (α) for the model was 0.05.

## Results

### Effects of HT on Plasma Membrane Integrity (SYBR-14/PI and PNA-FITC/PI)

**Figure 2:**
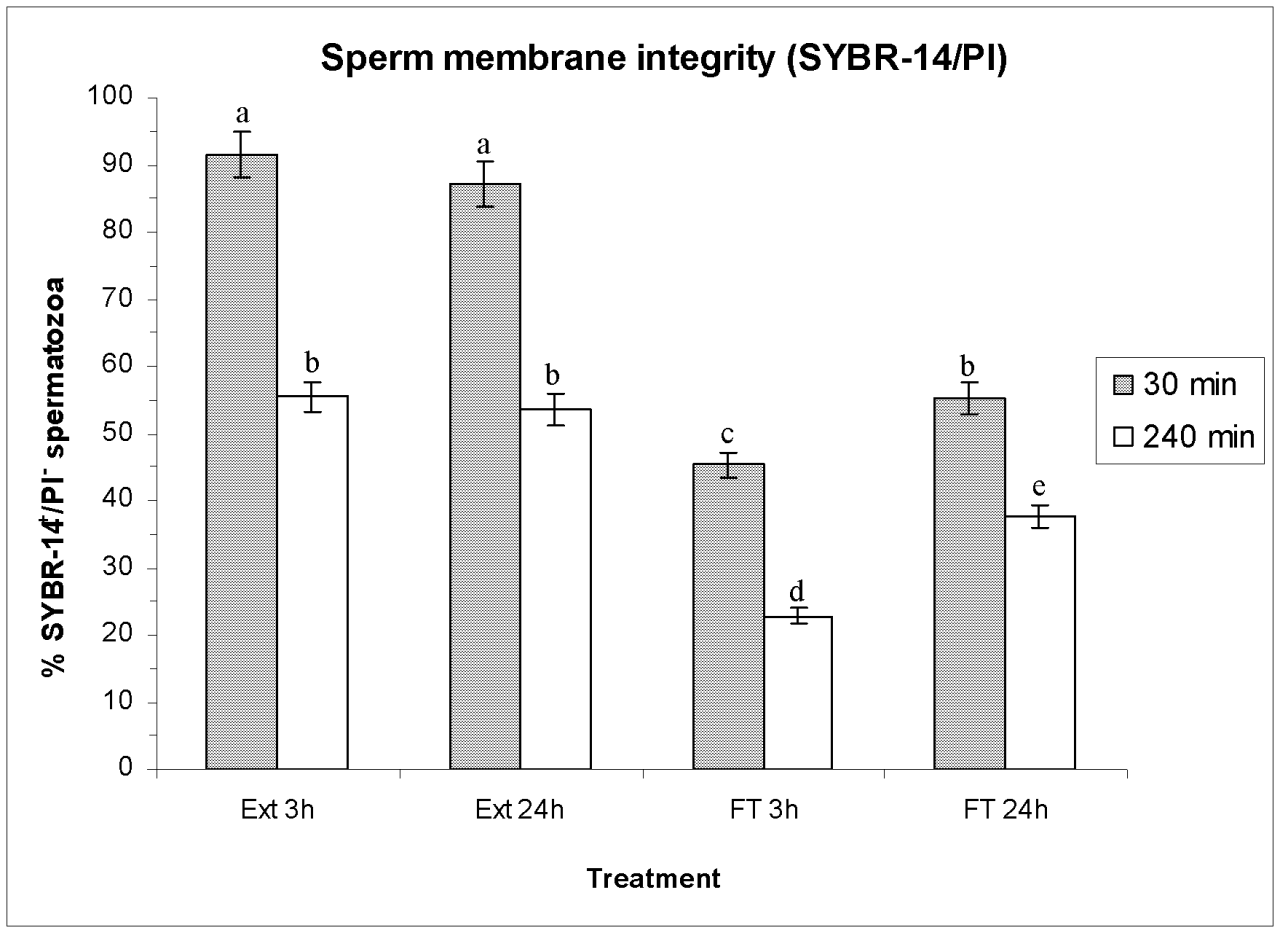
Percentage (as mean±SEM) of spermatozoa with intact plasma membrane (SYBR-14^+^/PI^−^, viable spermatozoa), before and after freeze/thawing when sperm is stored at 17°C either for 3 h or for 24 h. Different superscripts (*a*, *b*, *c*, *d*, *e*) mean significant differences (*P*<0.05) among bars, each bar representing different holding times (3 h or 24 h), preservation (Ext vs. F-T) and post-thawing times (30 min or 240 min). Ext = Extended semen (17°C). FT-C = Frozen-thawed semen.

No significant differences were observed in the plasma membrane integrity (SYBR-14/PI) of extended spermatozoa between both HTs (Ext 3 h *vs.* Ext 24 h; *P*>0.05) before freeze-thawing. Cryopreservation, instead, significantly (*P*<0.01) reduced the percentage of viable (SYBR-14^+^/PI^−^) spermatozoa (e.g. extended for 3 h: 91.5%±3.4% *vs.* FT 24 h: 53.1%±2.4%; *P*<0.001; means±SEM; see Figure 2). This decrease was significantly higher when the HT was of 3 h than when it was of 24 h (FT 3 h: 45.3%±1.9% vs. FT 24 h: 53.1%±2.4%; *P*<0.05). Incubation of the samples up to 240 min at 37°C also decreased the percentage of SYBR-14^+^/PI^−^ spermatozoa after freeze-thawing (e.g. extended for 24 h: 55.5%±2.3% *vs.* FT 24 h: 37.5%±1.7%; *P*<0.05), especially in the case of FT 3 h, whose reduction was again significantly higher than that of FT 24 h (FT 3 h: 22.9%±1.2% *vs*. FT 24 h: 37.5%±1.7%; *P*<0.05; see Figure 2).

**Figure 3:**
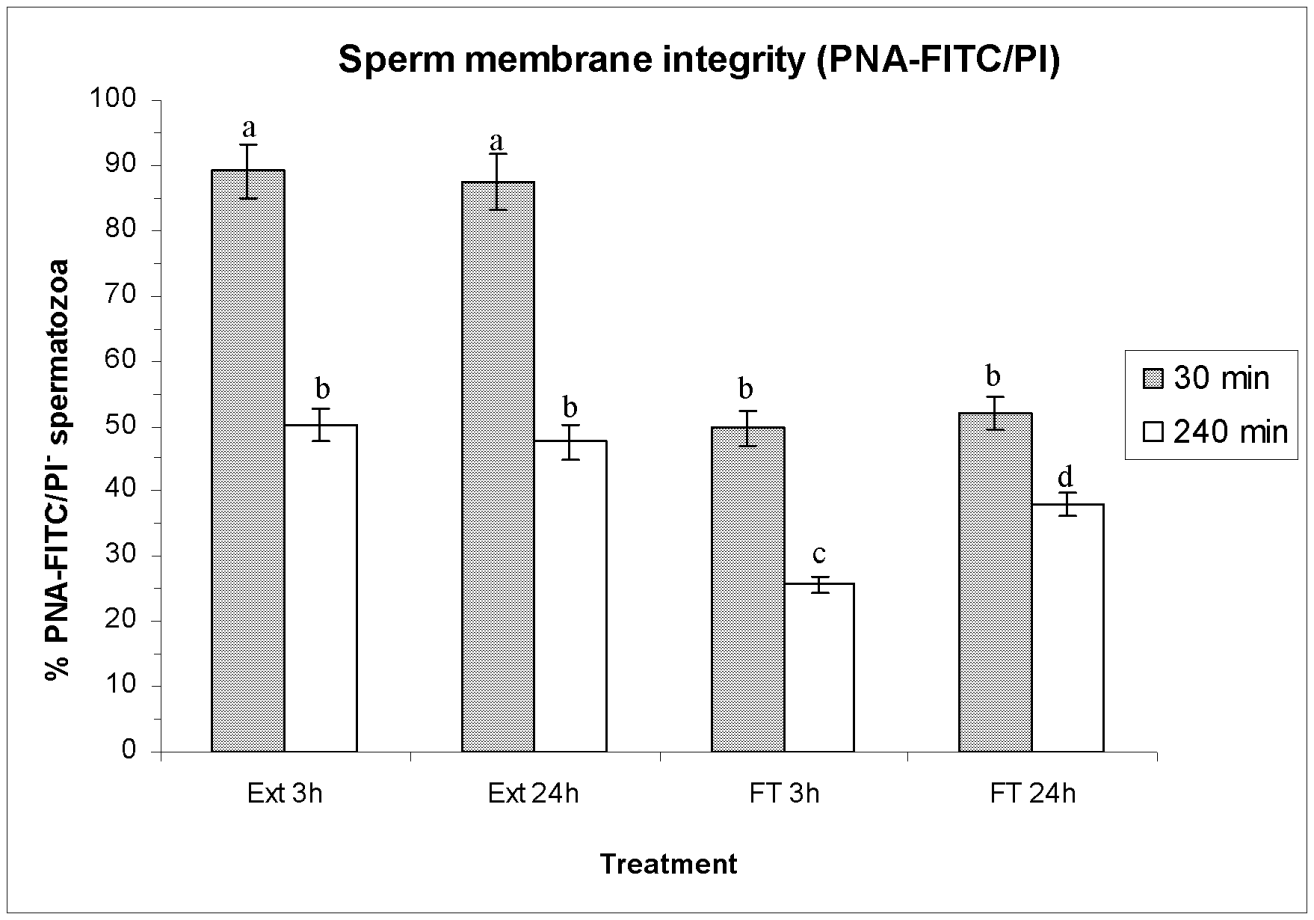
Percentages of spermatozoa with intact plasma membrane evaluated through PNA-FITC/PI assay. Bars show percentages of PNA-FITC^−/^PI^−^ spermatozoa (as mean±SEM) before and after freeze/thawing when sperm is stored at 17°C either for 3 h or for 24 h. Different superscripts (*a*, *b*, *c*, *d*) mean significant differences (*P*<0.05) among bars, each bar representing different holding times (3 h or 24 h), preservation (Ext vs. F-T) and post-thawing times (30 min or 240 min). Ext = Extended semen (17°C). FT-C = Frozen-thawed semen.

As far as PNA-FITC/PI staining is concerned, no significant differences between either HT were observed before freeze-thawing (Figure 3). In contrast, the percentages of spermatozoa with an intact plasma membrane (PNA-FITC^−/^PI^−^) were significantly lower in FT spermatozoa than in extended semen after 30 min and 240 min of incubation at 37°C, whereas PNA-FITC^+^/PI^+^ spermatozoa were significantly higher in the former (FT 3 h and FT 24 h) than in the latter (extended for 3 h and extended for 24 h; See Table S1). On the other hand, FT spermatozoa cryopreserved after a HT of 3 h presented a significantly (*P*<0.05) higher percentage of PNA-FITC^+^/PI^+^ spermatozoa than that cryopreserved after a HT of 24 h (See Table S1).

### Effects of HT on sperm membrane permeability (YO-PRO-1/PI)

**Figure 4:**
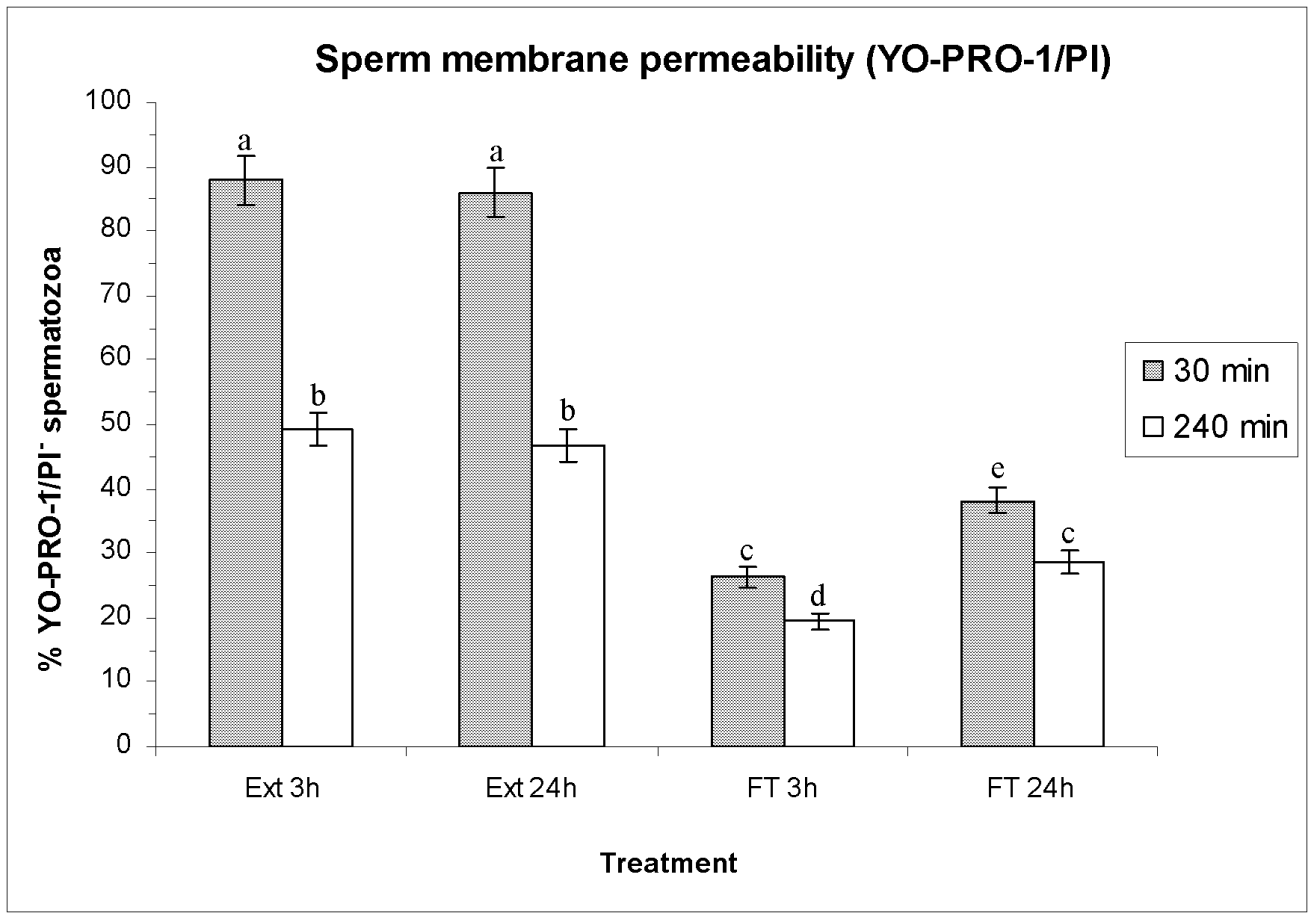
Sperm membrane permeability evaluated through YO-PRO-1/PI assay. Bars show percentages (as mean±SEM) of viable spermatozoa without changes in permeability of plasma membrane (YO-PRO-1^−/^PI^−^) before and after freeze/thawing when sperm is stored at 17°C either for 3 h or for 24 h. Different superscripts (*a*, *b*, *c*, *d*, *e*) mean significant differences (*P*<0.05) among bars, each bar representing different holding times (3 h or 24 h), preservation (Ext vs. F-T) and post-thawing times (30 min or 240 min). Ext = Extended semen (17°C). FT-C = Frozen-thawed semen.

Figure 4 shows, as means±SEM, the percentages of viable spermatozoa without changes in membrane permeability (YO-PRO-1^−/^PI^−^), whereas Table S2 also shows the percentages of viable spermatozoa with early changes in membrane permeability (YO-PRO-1^+^/PI^−^), and those of non-viable spermatozoa (PI^+^). Again, no significant differences were observed in sperm membrane permeability of extended sperm between both HTs before freeze-thawing. Freeze-thawing significantly decreased (*P*<0.05) the percentages of viable spermatozoa without changes in membrane permeability (Figure 4) and increased those of viable spermatozoa with early changes in membrane permeability and those of non-viable spermatozoa (Table S2). However, the extent of these changes differed in FT spermatozoa between HTs. Indeed, the decrease in viable spermatozoa without changes in membrane permeability observed after a HT of 3 h was significantly higher (*P*<0.05) than that observed after a HT of 24 h, both at 30 min and 240 min post-thawing (Figure 4).

### Effects of HT on Membrane Lipid Disorder (M540/YO-PRO-1)

**Figure 5:**
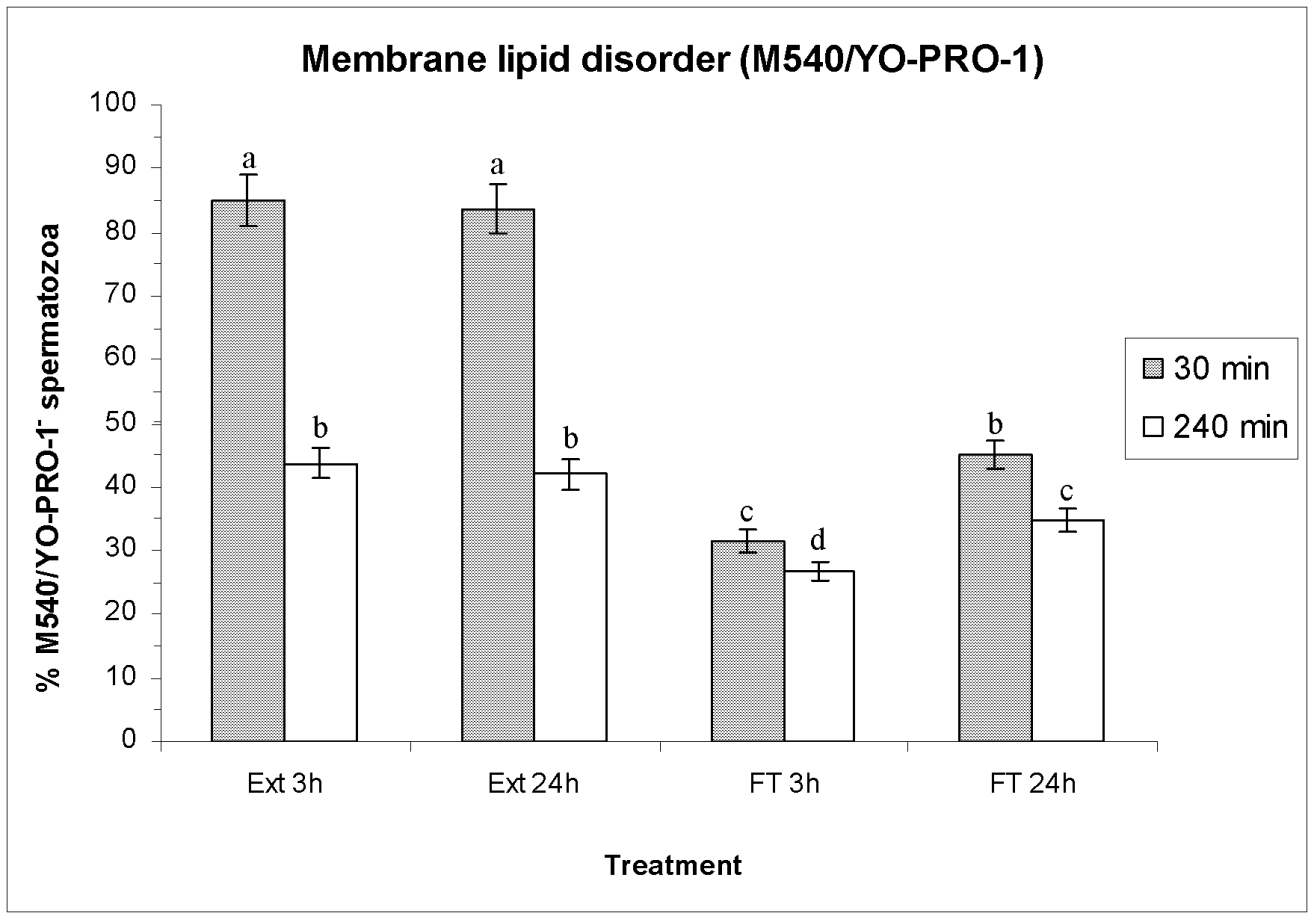
Sperm membrane lipid disorder evaluated through M540/YO-PRO-1 co-staining. The figure shows percentages (mean±SEM) of viable spermatozoa with low membrane lipid disorder (M540^−/^YO-PRO-1^−^) before and after freeze/thawing when sperm is stored at 17°C either for 3 h or for 24 h. Different superscripts (*a*, *b*, *c*, *d*, *e*) mean significant differences (*P*<0.05) among bars, each bar representing different holding times (3 h or 24 h), preservation (Ext vs. F-T) and post-thawing times (30 min or 240 min). Ext = Extended semen (17°C). FT-C = Frozen-thawed semen.

As in the previously described parameters, no significant differences were observed in membrane lipid disorder of extended spermatozoa between either HT before freeze-thawing (Figure 5). Freeze-thawing significantly increased (*P*<0.05) the percentages of non-viable spermatozoa with a high membrane lipid disorder (M540^+^/YO-PRO-1^+^) and decreased those of viable (M540^−/^YO-PRO-1^−^) and non-viable spermatozoa with a low lipid disorder (M540^−/^YO-PRO-1^+^), after 30 min and 240 min post-thawing (Figure 5; Table S3). Interestingly, the percentages of viable and non-viable spermatozoa with a high lipid disorder after freeze-thawing were significantly higher when the HT was of 3 h (FT 3 h) than when it was of 24 h (FT 24 h) (Table S3).

### Effects of HT on Intracellular Calcium Levels (Fluo3-AM/PI)

**Figure 6:**
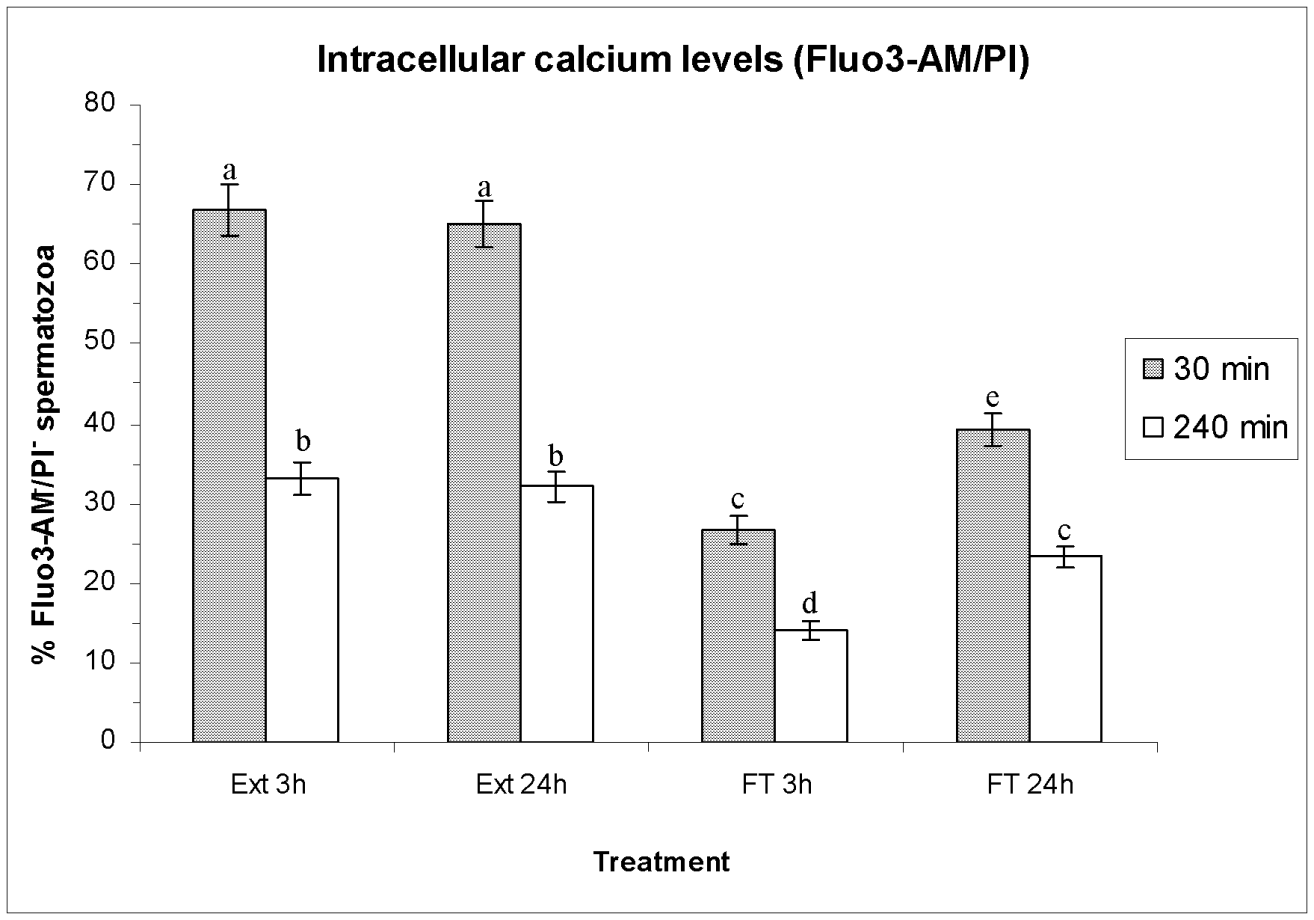
Intracellular calcium levels evaluated through Fluo3-AM/PI assay. Bars show percentages (as mean±SEM) of viable spermatozoa with low levels of intracellular calcium (Fluo3-AM^−/^PI^−^) before and after freeze/thawing when sperm is stored at 17°C either for 3 h or for 24 h. Different superscripts (*a*, *b*, *c*, *d*, *e*) mean significant differences (*P*<0.05) among bars, each bar representing different holding times (3 h or 24 h), preservation (Ext vs. F-T) and post-thawing times (30 min or 240 min). Ext = Extended semen (17°C). FT-C = Frozen-thawed semen.

Figure 6 shows, as means±SEM, the percentage of viable spermatozoa with low intracellular calcilum levels (Fluo3-AM^−/^PI^−^ spermatozoa), whereas Table S4 shows the percentages of viable and non-viable spermatozoa with high/low levels of intracellular calcium and the geometric mean of Fluo3^+^. In a similar fashion to the previously described parameters, no significant differences were observed in sperm intracellular calcium levels of extended spermatozoa between either HT before freeze-thawing (Figure 6, Table S4). Both after 30 min and 240 min of incubation at 37°C, the geometric mean of Fluo3^+^ intensity was significantly lower in FT spermatozoa than in extended semen. Freeze-thawing also decreased the percentages of viable spermatozoa with low and high levels of intracellular calcium (Fluo3^−/^PI^−^ and Fluo3^+^/PI^−^) and increased those of non-viable spermatozoa with low levels of intracellular calcium (Fluo3^−/^PI^+^), but the extent of these changes differed between HTs. Indeed, the percentages of non-viable spermatozoa with low levels of intracellular calcium were significantly higher (*P*<0.05) in the sperm cryopreserved after a HT of 3 h than in that cryopreserved after a HT of 24 h, both after 30 min and 240 min post-thawing (See Table S4).

### Effects of HT on ROS Levels (H_2_DFCDA/PI and HE/YO-PRO-1)

**Figure 7:**
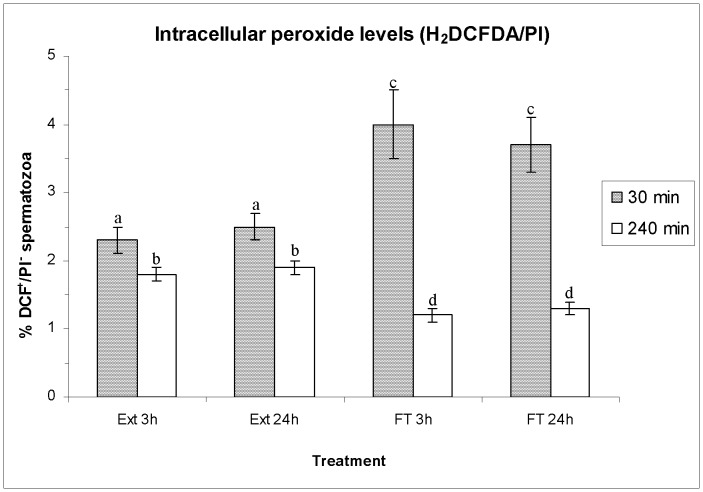
Intracellular peroxide levels (H_2_DCFDA/PI staining), as percentages (as mean±SEM) of viable spermatozoa with high levels of intracellular peroxides (DCF^ +^/PI^−^) before and after freeze/thawing when sperm is stored at 17°C either for 3 h or for 24 h. Different superscripts (*a*, *b*, *c*, *d*, *e*) mean significant differences (*P*<0.05) among bars, each bar representing different holding times (3 h or 24 h), preservation (Ext vs. F-T) and post-thawing times (30 min or 240 min). Ext = Extended semen (17°C). FT-C = Frozen-thawed semen.

Figure 7 (mean±SEM) shows percentages of viable spermatozoa with high peroxide levels (DCF^+^/PI^−^) evaluated through H_2_DFCDA/PI co-staining, before and after boar-sperm cryopreservation. Other parameters evaluated by H_2_DFCDA/PI assay are shown in Table S5. Both percentages of spermatozoa DCF^+^/PI^−^ (Figure 7) and the geometric mean of DCF^+^-intensity in viable spermatozoa (Table S5) were significantly lower in FT than in extended spermatozoa, although no significant differences between HTs were found either in FT or in extended spermatozoa (Figure 7). In addition, the geometric mean of DCF^+^ in total spermatozoa was significantly higher in FT (FT 3 h and FT 24 h) than in extended spermatozoa (extended for 3 h and extended for 24 h), but no significant differences between HT were found (Table S5).

As far as the superoxide levels are concerned (HE/YO-PRO-1 co-staining), boar-sperm cryopreservation did not affect either the percentage of E^+^/YO-PRO-1^−^ or the geometric mean of E^+^ intensity in total spermatozoa. In contrast, the geometric mean of E^+^-intensity in the viable sperm population was significantly higher in the extended rather than in the FT spermatozoa, but significant differences were not observed between either HT (Table S6).

### Effects of HT on the Amounts of Free Cysteine Residues in Sperm Nucleoproteins

**Figure 8:**
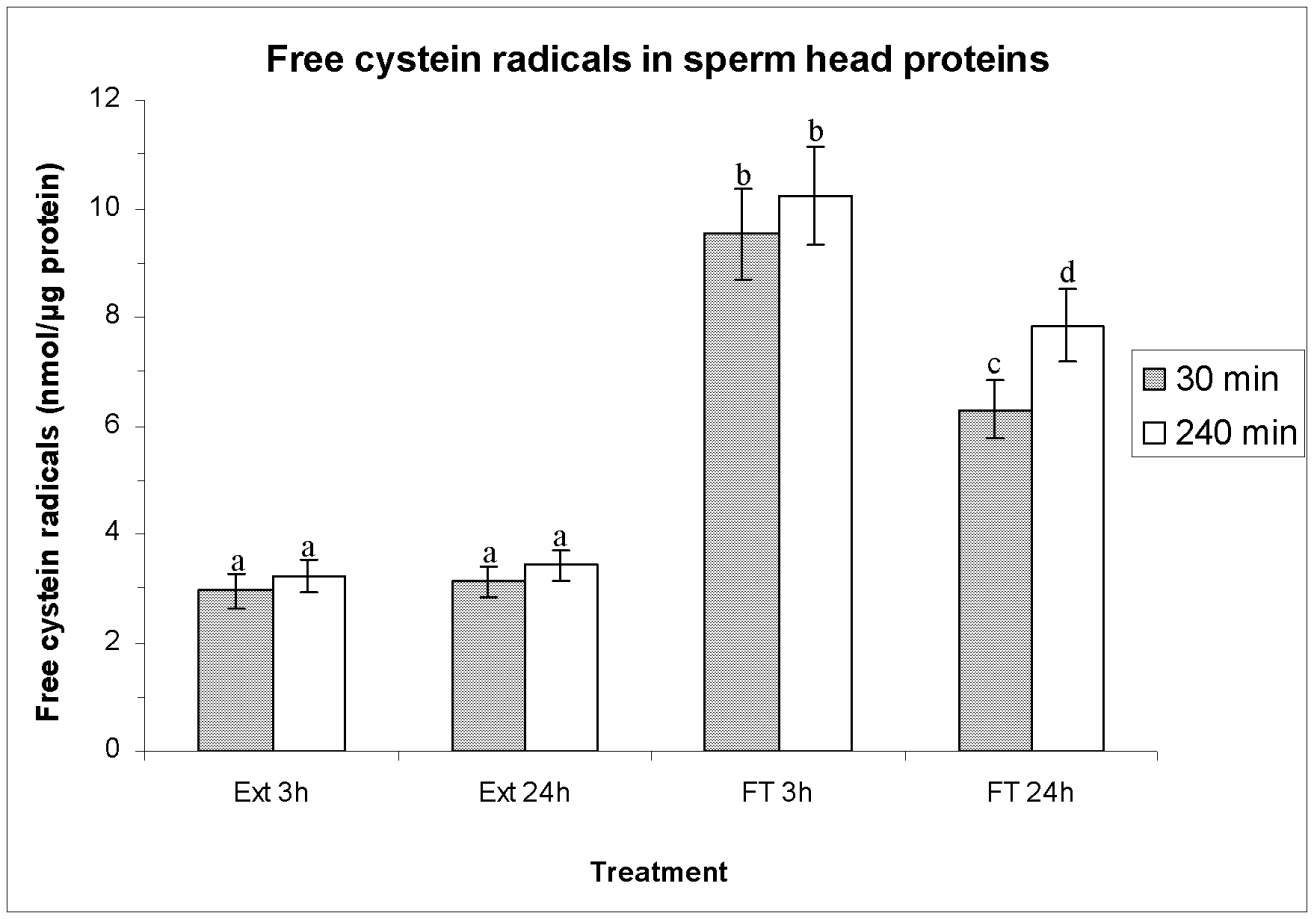
Free cysteine radicals (FCR) in sperm-head proteins (as mean±SEM), before and after freeze-thawing when sperm is stored at 17°C either for 3 h or for 24 hours. Different superscripts (*a*, *b*, *c*, *d*) mean significant differences (*P*<0.05) among bars, each bar representing different holding times (3 h or 24 h), preservation (Ext vs. F-T) and post-thawing times (30 min or 240 min). Ext = Extended semen (17°C). FT-C = Frozen-thawed semen.

Before freeze-thawing, the levels of free cysteine radicals in sperm nucleoproteins were similar in both HTs (Figure 8; i.e. extended for 3 h: 3.0 nmol·µg protein^−1^±0.3 nmol·µg protein^−1^ *vs.* extended for 24 h: 3.1 nmol·µg protein^−1^±0.3 nmol·µg protein^−1^ after 30 min of incubation at 37°C). In contrast, sperm cryopreservation significantly increased (*P*<0.01) the levels of free cysteine radicals, as compared to extended semen. This increase was significantly higher when the HT was 3 h rather than when it was 24 h after both 30 min and 240 min post-thawing (e.g. FT 3 h: 9.5 nmol·µg protein^−1^±0.8 nmol·µg protein^−1^ *vs.* FT 24 h: 6.3 nmol·µg protein^−1^±0.5 nmol·µg protein^−1^, after 30 min of thawing).

### Effects of HT on Sperm DNA Fragmentation

**Figure 9:**
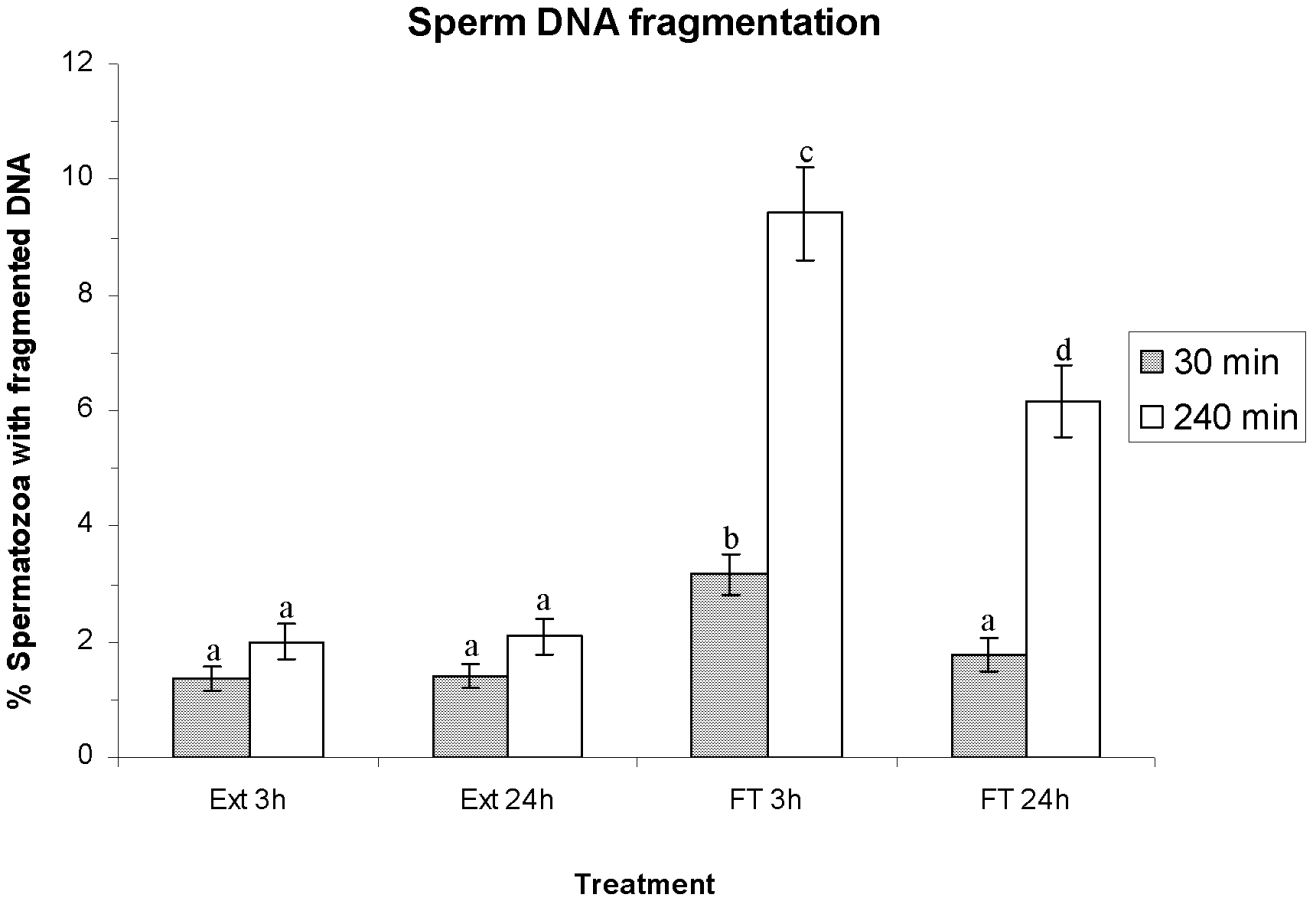
Sperm DNA fragmentation (SDF) as percentage of spermatozoa with fragmented DNA (mean±SEM), before and after freeze/thawing when sperm is stored at 17°C either for 3 h or for 24 h. Different superscripts (*a*, *b*, *c*, *d*) mean significant differences (*P*<0.05) among bars, each bar representing different holding times (3 h or 24 h), preservation (Ext vs. FT) and post-thawing times (30 min or 240 min). Ext = Extended semen (15°C). FT = Frozen-thawed semen.

Figure 9 shows the percentage of spermatozoa with fragmented DNA as means±SEM. No significant differences between HTs were observed in the percentages of spermatozoa with fragmented DNA before freeze-thawing (e.g. extended for 3 h: 1.3%±0.2%; extended for 24 h: 1.4%±0.2%; *P*>0.05 after incubation at 37°C for 30 min). Percentages of spermatozoa with fragmented DNA in extended semen did not differ (*P*>0.05) from those obtained in FT spermatozoa cryopreserved after a HT of 24 h at 30 min post-thawing (FT 24 h: 1.8%±0.2%). Conversely, the percentages of spermatozoa with fragmented DNA in FT spermatozoa cryopreserved after a HT of 3 h (FT 3 h: 3.2%±0.4%) were significantly higher (*P*<0.05) than those observed in the other cases (i.e. Ext 3 h, Ext 24 h, FT 24 h) at 30 min post-thawing.

After 240 min post-thawing, the percentages of spermatozoa with fragmented DNA in FT spermatozoa (FT 3 h and FT 24 h) were significantly higher (*P*<0.05) than those observed in extended semen (Ext 3 h: 2.0%±0.3%, and Ext 24 h: 2.1%±0.3%). Again, the percentages of spermatozoa with fragmented DNA in FT spermatozoa cryopreserved after a shorter HT were significantly higher than those cryopreserved after a longer HT (FT 3 h: 9.4%±0.8% vs. FT 24 h: 6.2%±0.6%; *P*<0.05).

### Effects of HT on Sperm Motility

Cryopreservation significantly decreased (*P*<0.05) in all cases (extended *vs.* FT) the percentages of total and progressive motile spermatozoa and VCL and VAP (Table 1). This reduction was observed both after 30 min and 240 min post-thawing. On the other hand, and after 240 min post-thawing, FT spermatozoa cryopreserved at a HT of 24 h presented significantly (*P*<0.05) higher values of %TMOT, %PMOT, VCL and VAP than FT spermatozoa cryopreserved at a HT of 3 h.

**Table 1:** Effects of holding time prior to freeze-thawing on different sperm motility parameters after 30 and 240 min post-thawing at 37°C.

	*Extended 3* *h*		*Extended 24* *h*		*Frozen-Thawed 3* *h*		*Frozen-Thawed 24* *h*	
	*30 min*	*240 min*	*30 min*	*240 min*	*30 min*	*240 min*	*30 min*	*240 min*
**% TMOT**	87.2±4.8^a^	58.4±2.9^b^	86.6±4.9^a^	57.5±2.8^b^	48.4±2.9^b^	26.7±1.6^e^	55.6±3.0^b, c^	36.9±2.0^d^
**%PMOT**	65.3±3.2^a^	38.6±2.3^b^	64.2±3.1^a^	37.8±2.2^b^	30.1±1.8^c^	14.8±0.9^e^	34.5±2.3^b, c^	21.2±1.1^d^
**VSL (µm·s** ^−**1**^ **)**	26.6±2.4^a^	23.8±2.3^a, b^	27.4±2.3^a^	22.5±2.2^a, b^	19.1±2.0^b, c^	14.1±1.5^c^	23.1±2.5^a, b^	19.4±1.9^b, c^
**VCL (µm·s** ^−**1**^ **)**	53.9±3.2^a^	38.4±2.1^b^	54.1±3.1^a^	38.2±2.2^b^	34.3±2.3^b, c^	23.6±1.8^d^	40.9±2.4^b^	31.0±2.4^c^
**VAP (µm·s** ^−**1**^ **)**	36.4±2.5^a^	28.8±1.7^b^	34.6±2.3^a^	28.6±1.6^b, c^	25.7±1.8^c^	17.8±1.4^d^	31.8±2.5^a, b^	24.2±1.7^c^
**LIN (%)**	49.3±2.9^a^	62.4±3.6^b^	49.7±2.8^a^	62.1±3.6^b^	56.8±3.4^b^	60.1±3.9^b^	56.5±3.1^b^	59.5±3.5^b^
**STR (%)**	73.3±3.6^a^	78.1±3.9^a^	79.8±3.6^a^	77.2±3.9^a^	74.2±3.5^a^	78.6±3.8^a^	73.0±3.5^a^	80.2±3.8^a^
**WOB (%)**	68.5±3.8^a^	75.2±4.1^a^	67.0±3.7^a^	74.8±4.3^a^	74.9±4.1^a^	75.8±4.2^a^	76.1±4.3^a^	75.6±4.0^a^

Data are shown as mean ± SEM. Different superscripts (*a, b, c*, *d*) mean significant differences (*P*<0.05) within rows (i.e. for a given motility parameter) among columns (i.e. comparing extended and frozen-thawed semen, and 30 and 240 min of post-thawing incubation at 37°C).

### Cryotolerance Indexes and Principal Component Analyses

Principal component analyses using cryotolerance indexes are shown in Tables 2 and 3. In the case of cryotolerance indexes calculated after 30 min of incubation at 37°C (Table 2), a total of five components were extracted and the explained variance was 91.23%. The first component explained a variance of 59.49%, and included the most important cryotolerance indexes describing sperm integrity and survival (i.e. viable spermatozoa with no alterations in membrane permeability, %TMOT, %PMOT, levels of free cysteine radicals in sperm nucleoproteins…). In contrast, cryotolerances indexes of peroxides and superoxide levels were included in the other four components.

**Table 2:** Principal component analysis of sperm cryotolerance with parameters (x) evaluated after incubation at 37°C for 30 min.

Component	Variance	Combinations of variables	a_ij_	a_ij_^2^
1	59.49%	% Viable spermatozoa (SYBR-14^+^/PI^−^)	0.92	0.85
		Free cysteine radicals in sperm nucleoproteins	−0.91	0.83
		% Spermatozoa with fragmented DNA	−0.89	0.79
		% Non-viable spermatozoa with high lipid disorder (M540^+^/PI^+^)	−0.85	0.72
		% Viable spermatozoa without changes in m.p. (YO-PRO-1^−/^PI^−^)	0.85	0.72
		% Membrane intact spermatozoa (PNA-FITC^−/^PI^−^)	0.84	0.71
		% Viable spermatozoa with low lipid disorder (M540^−/^PI^−^)	0.82	0.67
		% TMOT	0.81	0.66
		% Non-viable spermatozoa (YO-PRO-1^+/−/^PI^+^)	−0.81	0.66
		% Viable spermatozoa with high lipid disorder (M540^+^/PI^−^)	−0.80	0.64
		% Membrane damaged spermatozoa that present o.a.m. (PNA-FITC^+^/PI^+^)	−0.80	0.64
		VAP	0.79	0.62
		% Viable spermatozoa with low intracellular Ca^2+^ levels (Fluo3-AM^−/^PI^−^)	0.79	0.62
		VCL	0.78	0.61
		VSL	0.77	0.59
		% Viable spermatozoa with early changes in m.p. (YO-PRO-1^+^/PI^−^)	−0.75	0.56
		% Non-viable spermatozoa with low intracellular Ca^2+^ levels (Fluo3-AM^−/^PI^+^)	−0.71	0.50
		% PMOT	0.70	0.49
		GMFI Fluo3-AM^+^	−0.64	0.41
2	11.95%	% Membrane damaged spermatozoa with lost o.a.m. (PNA-FITC^−/^PI^+^)	−0.86	0.74
		% Viable spermatozoa with high intracellular Ca^2+^ levels (Fluo3-AM^+^/PI^−^)	−0.67	0.45
		% Viable spermatozoa with high intracellular peroxide levels (DCF^+^/PI^−^)	0.59	0.35
		Non-viable spermatozoa with high intracellular Ca^2+^ levels (Fluo3-AM^+^/PI^+^)	−0.57	0.32
3	8.30%	GMFI E^+^/PI^−^	0.84	0.71
		GMFI E^+^/Total	−0.64	0.41
4	6.78%	GMFI DCF^+^/PI^−^	0.98	0.96
		GMFI DCF^+^/Total	0.68	0.46
5	4.71%	% LIN	0.87	0.76
		% STR	0.79	0.62
		% WOB	−0.62	0.38
**Total**	**91.23%**			

Each cryotolerance index was calculated as follows: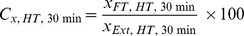.

(m.p.: membrane permeability; o.a.m.: outer acrosome membrane; GMFI: Geometric mean of fluorescence intensity; arbitrary units).

**Table 3:** Principal component analysis of sperm cryotolerance with parameters (x) evaluated after incubation at 37°C for 240 min.

Component	Variance	Combinations of variables	a_ij_	a_ij_^2^
1	64.11%	% Viable spermatozoa without changes in m.p. (YO-PRO-1^−/^PI^−^)	0.95	0.90
		Free cysteine radicals in sperm nucleoproteins	−0.95	0.90
		% Spermatozoa with fragmented DNA	−0.94	0.88
		% Non-viable spermatozoa (YO-PRO-1^+/−/^PI^+^)	−0.93	0.86
		% Viable spermatozoa (SYBR-14^+^/PI^−^)	0.91	0.83
		% Viable spermatozoa with low intracellular Ca^2+^ levels (Fluo3-AM^−/^PI^−^)	0.88	0.77
		% Viable spermatozoa with low lipid disorder (M540^−/^PI^−^)	0.86	0.74
		% TMOT	0.83	0.69
		% Non-viable spermatozoa with low intracellular Ca^2+^ levels (Fluo3-AM^−/^PI^+^)	−0.82	0.67
		% Viable spermatozoa with high lipid disorder (M540^+^/PI^−^)	−0.82	0.67
		% Membrane intact spermatozoa (PNA-FITC^−/^PI^−^)	0.81	0.66
		VAP	0.79	0.62
		VCL	0.77	0.59
		% Non-viable spermatozoa with high lipid disorder (M540^+^/PI^+^)	−0.76	0.58
		% Membrane damaged spermatozoa that present o.a.m. (PNA-FITC^+^/PI^+^)	−0.75	0.56
		VSL	0.75	0.56
		GMFI E^+^/YO-PRO-1^−^	0.74	0.55
		% PMOT	0.72	0.52
		% Viable spermatozoa with early changes in m.p. (YO-PRO-1^+^/PI^−^)	−0.66	0.44
		% Membrane damaged spermatozoa with lost o.a.m. (PNA-FITC^−/^PI^+^)	−0.66	0.44
		% Non-viable spermatozoa with low membrane lipid disorder (M540^−/^PI^+^)	0.63	0.40
2	11.49%	GMFI E^+^/Total	−0.90	0.80
		% Viable spermatozoa with high intracellular superoxide levels (E^+^/YO-PRO-1^−^)	−0.86	0.73
		% Viable spermatozoa with high intracellular peroxide levels (DCF^+^/PI^−^)	−0.75	0.56
		% Viable spermatozoa with high intracellular calcium levels (Fluo3-AM^+^/PI^−^)	0.71	0.51
3	9.70%	GMFI DCF^+^/PI^−^	0.96	0.92
		GMFI DCF^+^/Total	0.80	0.64
		% LIN	0.77	0.59
		% STR	0.62	0.38
4	5.24%	GMFI Fluo3-AM^+^	−0.91	0.83
5	3.66%	% WOB	−0.89	0.79
**Total**	**94.20%**			

Each cryotolerance index was calculated as follows: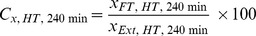.

(m.p.: membrane permeability; o.a.m.: outer acrosome membrane; GMFI: Geometric mean of fluorescence intensity; arbitrary units).

In a similar fashion to the last case, five components were also obtained from PCA with cryotolerance indexes evaluated after 240 min of thawing at 37°C (Table 3). The total explained variance was of 94.20% and, again, the first component represented more than a half of total variance (64.11%) and included the most significant crytolerance indexes describing sperm integrity and survival.

### Mini-array Analyses of Serine-phosphorylation Levels of 30 Proteins Involved in the Overall Regulation of Boar-sperm Function

**Figure 10:**
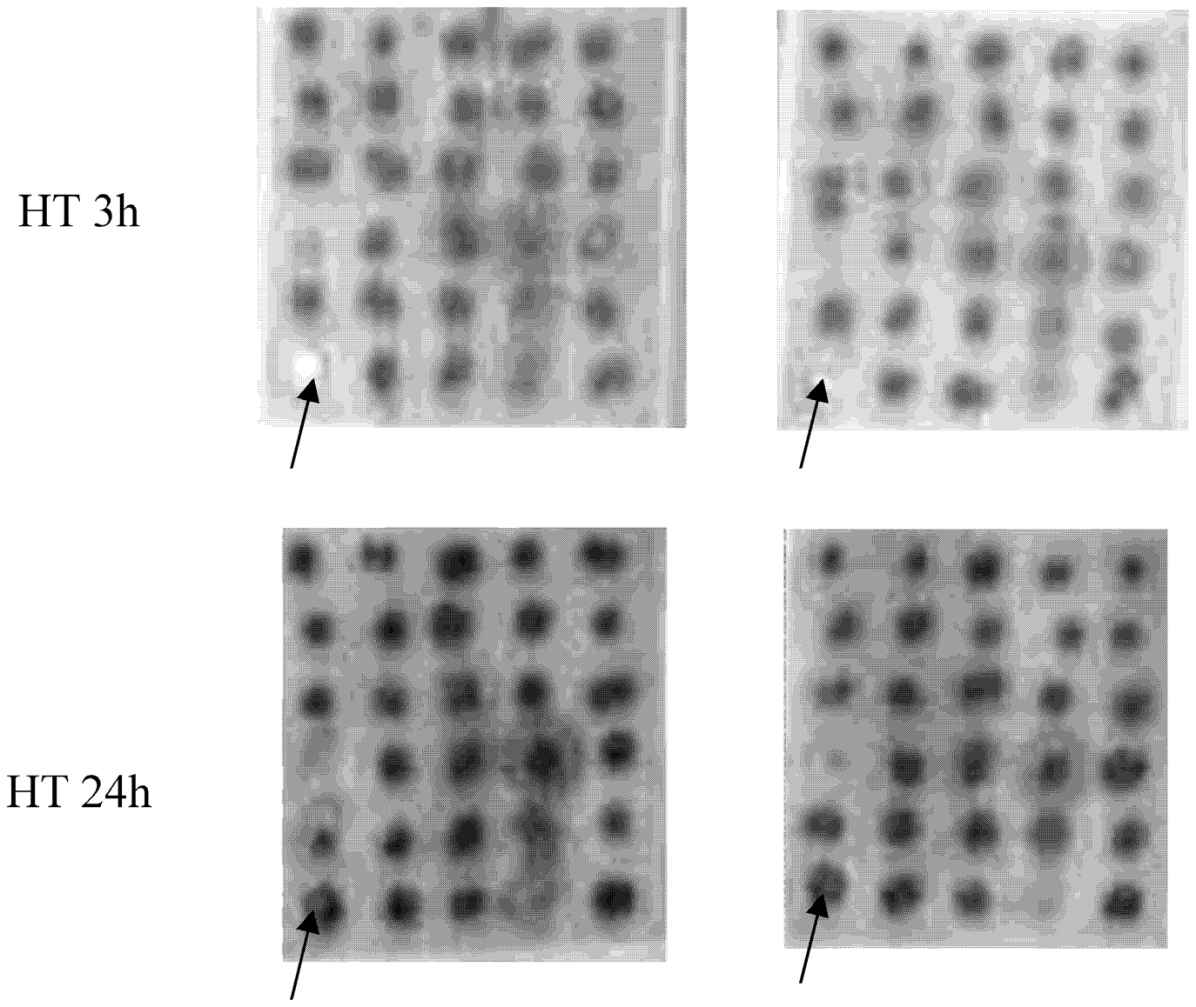
Mini-array analysis of the serine phosphorylation status of 30 sperm proteins after a HT of 3 h or 24 h. The mini-array analysis and the distribution of the proteins analysed are described in Figure 1. The figure shows a representative image of two replicates coming from two different sperm samples and is representative of twelve separate experiments. Arrows (→) mark HSP70.

Figure 10 and Table 4 show the changes in pSer levels of 30 different proteins after both HTs (3 h and 24 h). Storing sperm samples for a longer HT (24 h) resulted in a significant increase (*P*<0.05) increase in the phosphorylation levels of serine residues in HSP70, TRK(A, B and C), CDK1/CDC2 and GSK-3α, when compared with the phosphorylation levels of serine residues after a shorter HT (3 h). Notwithstanding, from these four proteins, the highest increase was seen in HSP70, which increased pSer levels from 100.0±0.0 arbitrary units (3 h) to 150.2±5.1 arbitrary units (24 h).

**Table 4:** Comparison of serine-phosphorylation levels in 30 different sperm proteins after assessment through mini-array analyses.

*Proteins*	*Holding time at 17*°*C*	
	3 h (arbitrary units)	24 h (arbitrary units)
**AKT-1/AKT-2**	100.0±0.0^a^	100.5±2.0^a^
**CDK6**	100.0±0.0^a^	99.8±1.9^a^
**CYCLIN E**	100.0±0.0^a^	108.6±3.2^a^
**IRAK**	100.0±0.0^a^	106.3±3.7^a^
**PYK2/CAKβ**	100.0±0.0^a^	109.4±2.6^a^
**CASPASE 9**	100.0±0.0^a^	104.8±2.5^a^
**c-kit**	100.0±0.0^a^	105.3±2.0^a^
**CYCLIN H**	100.0±0.0^a^	104.2±2.3^a^
**PI3 KINASE/p85**	100.0±0.0^a^	109.8±3.4^a^
**C-RAF-1**	100.0±0.0^a^	103.0±4.1^a^
**CDC25**	100.0±0.0^a^	103.2±2.5^a^
**ERK-1**	100.0±0.0^a^	110.9±1.4^a^
**PKC**	100.0±0.0^a^	108.4±2.0^a^
**RAS**	100.0±0.0^a^	106.9±2.8^a^
**CDC6**	100.0±0.0^a^	110.3±2.4^a^
**CLUSTERIN**	100.0±0.0^a^	102.7±2.8^a^
**ERK-2**	100.0±0.0^a^	112.2±2.9^a^
**PP1, PP2A, PP2B, PPX**	100.0±0.0^a^	109.5±3.2^a^
**TRK Total (A, B, C)**	100.0±0.0^a^	120.8±3.4^b^
**CDK1/CDC2**	100.0±0.0^a^	120.7±2.1^b^
**CLYCLIN A**	100.0±0.0^a^	109.1±4.1^a^
**GSK-3α**	100.0±0.0^a^	125.6±0.9^b^
**PTP1 (SH)**	100.0±0.0^a^	107.5±2.5^a^
**CDK2**	100.0±0.0^a^	109.9±1.5^a^
**CYCLIN B**	100.0±0.0^a^	102.4±2.6^a^
**HSP70**	100.0±0.0^a^	150.2±5.1^b^
**PTP1B**	100.0±0.0^a^	112.6±2.9^a^
**CDK4**	100.0±0.0^a^	107.6±2.7^a^
**CYCLIN D3**	100.0±0.0^a^	105.2±5.5^a^
**PTP2 (SH)**	100.0±0.0^a^	107.5±1.9^a^

The values obtained for the HT of 3 h were transformed in order to obtain a basal arbitrary value of 100, from which the intensity values for the other samples (i.e. HT 24 h) were calculated. Results are given as mean ± SEM, and different superscripts (*a*, *b*) mean significant differences (*P*<0.05) within rows (i.e. between the two holding times) for each protein.

### Specific Immunopreciptation Analysis of pSer Changes of HSP70 during HT

**Figure 11:**
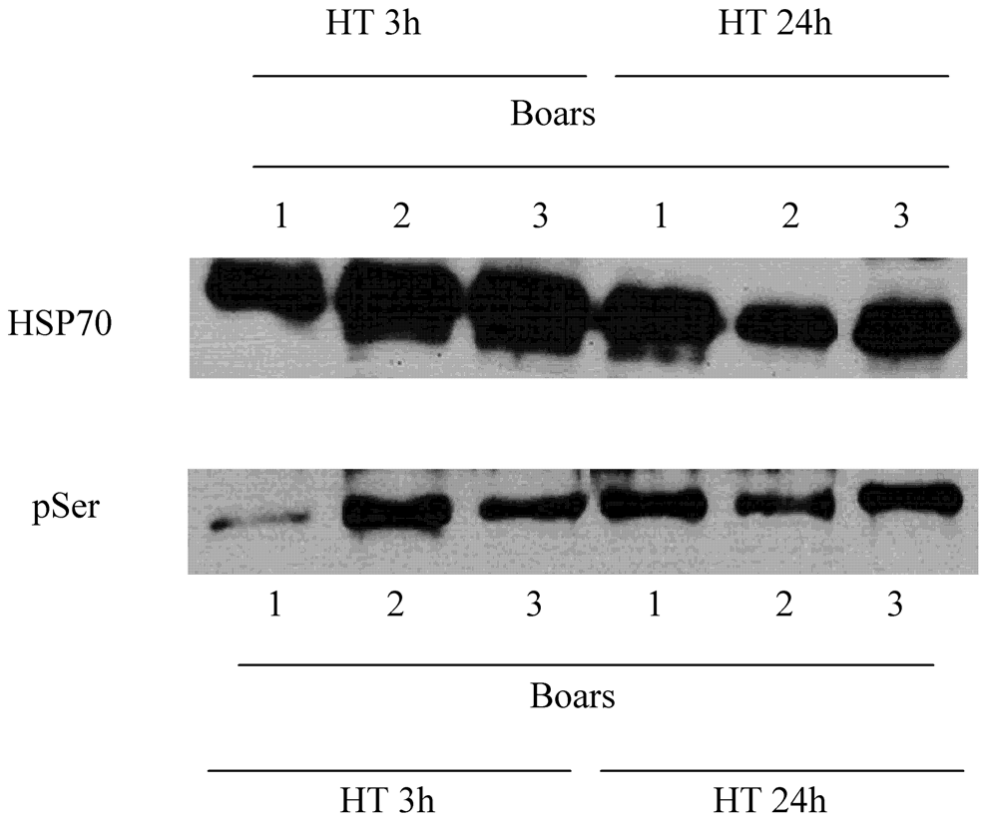
Representative Western-blots against HSP70 and pSer in immunoprecipitation studies after a HT of 3 h or 24 h. Three replicates coming from three different sperm samples are shown. The figure is representative of twelve different sperm samples stored either for 3 h or 24 h.

The specific immunoprecipitation analysis confirmed that pSer of HSP70 was specifically and significantly higher (*P*<0.05) when the HT was of 24 h than when it was of 3 h (Figure 11). Indeed, the ratio between serine phosphorylated HSP70 and total content of HSP70 protein (pSer-HSP70:total HSP70) was significantly higher (*P*<0.05) after a HT of 24 h (0.68±0.05) than after a HT of 3 h (0.35±0.03).

### Multiple Regression Analyses between pSer in HSP70 and Cryotolerance Indexes

Table 5 shows Pearson correlation coefficients between extracted components from PCA with cryotolerances indexes (calculated after incubation for 30 or 240 min at 37°C) and values from pSer levels in HSP70 obtained in mini-array analyses and in immuno-precipitation against HSP70 assessment (pSer-HSP70:total HSP70 ratio). Whereas the first components from PCA obtained from cryotolerance indexes, both at 30 and 240 min of incubation at 37°C, were positively and significantly correlated with pSer levels in HSP70 obtained in mini-array analyses and pSer-HSP70:total HSP70 ratios observed after immunoprecipitation, no significant correlation was seen for the other four components.

**Table 5:** Pearson correlation coefficients between the extracted components from PCA with cryotolerances indexes (calculated after incubation for 30 or 240 min at 37°C) and values from pSer levels in HSP70 obtained in mini-array analyses and in immuno-precipitation against HSP70 assessment (pSer-HSP70:total HSP70 ratio) (*P<0.05).

		HSP70 (miniarray values)	pSer-HSP70:total HSP70 ratio (immunoprecipitation)
**30 min**	Regression factor for Component 1	0.77*	0.74*
	Regression factor for Component 2	−0.33	−0.29
	Regression factor for Component 3	−0.02	−0.08
	Regression factor for Component 4	−0.05	−0.08
	Regression factor for Component 5	0.16	0.15
**240 min**	Regression factor for Component 1	0.75*	0.76*
	Regression factor for Component 2	0.35	0.31
	Regression factor for Component 3	0.06	−0.08
	Regression factor for Component 4	−0.04	0.10
	Regression factor for Component 5	−0.08	0.04

Given that the relevance of the first component in sperm cryotolerance, such component was used in regression equations as an independent variable, whereas dependent variables submitted to the stepwise forward model were all the sperm parameters (not cryotolerance indexes but raw sperm parameters) evaluated before freeze-thawing. As dependent variable, spots intensity for each protein obtained in mini-array protein analyses and pSer-HSP70:total HSP70 ratio were also included and results are shown in Table 6. From all the dependent variables submitted to the model, only the pSer-HSP70:total HSP70 ratio was included, whereas the others were left out. In all the cases, up to four models were run and all included the same dependent variable. Thus, predicting sperm cryotolerance before freeze-thawing resulted to be possible when considering the pSer-HSP70:total HSP70 ratios but not the other sperm parameters.

**Table 6:** Regression equations between regression component 1 of PCA with cryotolerance indexes (*y*) and sperm parameters evaluated before freeze-thawing.

	Regression equation	R^2^	R	*P* value model
***30 min***				
Lineal	y = 4.61x−2.46	0.56	0.75	<0.05
Logarithmic	y = 2.34ln(x)+1.60	0.55	0.74	<0.05
Inverse	y = −1.19/x+2.42	0.56	0.75	<0.05
Quadratic	y = −7.74x^2^+11.57x−0.57	0.59	0.77	<0.05
***240 min***				
Lineal	y = 4.76x−2.54	0.58	0.76	<0.01
Logarithmic	y = 2.46ln(x)+1.65	0.58	0.76	<0.01
Inverse	y = −1.23/x+2.51	0.59	0.77	<0.01
Quadratic	y = −1.65x^2^+5.99x−0.97	0.58	0.76	<0.05

From all the dependent variables submitted to the model, only the pSer-HSP70:total HSP70 ratio (*x*) was included, whereas the others were left out.

## Discussion

Our results confirm that HT for 24 h has an improving effect on boar-sperm cryotolerance. In addition, we have also shown that this improving effect is concomitant with changes in pSer levels of proteins like HSP70. This change suggests that the improving action of HT could be related to post-transcriptional changes in the activity of the anti-stress molecular mechanisms launched by boar spermatozoa during freeze-thawing, taking into account that spermatozoa are not able to up/downregulate their gene expression. In this way, covalent post-translation protein-modification mechanisms like protein phospho/dephosphorylation [9], [11] will be one of the most important post-translational mechanisms involved in the modulation of these anti-stress mechanisms.

In the present study, boar-sperm cryopreservation has been utilised as a model for inducing stressful conditions [1]. However, this application required a previous approach in order to better learn the effects of HT on the success of sperm cryopreservation. Thus, the absence of significant differences between HTs in any of the sperm functional parameters before starting the cryopreservation (i.e. in extended semen) is noteworthy. In contrast, the existence of differences between HTs after freeze-thawing indicates that during this storage period boar spermatozoa increase their cryotolerance. These conclusions are only partially supported by the existing literature, since there are only a few reports regarding the HT effects on boar-sperm post-thaw quality and their results are controversial [5]–[7]. In addition, these effects have mainly been reported so far on the basis of post-thaw sperm viability and motility assessments, without evaluating other relevant parameters such as the stability of the sperm nucleus or intracellular ROS levels. Against this background, the present work clearly demonstrates that a HT of 24 h prior to sperm cryopreservation deteriorates the post-thawed sperm cell less than does a HT of 3 h.

In the case of the sperm-nucleus structural integrity, our results indicate for the first time that the extent of damages that freeze-thawing infringes upon the boar-sperm nucleus depends on the HT period. It is worth mentioning that previous studies of our group have observed that freeze-thawing increases the number of disrupted disulphide bonds in boar-sperm nucleoproteins [34], [45]. Despite the mechanism which underlies the rupture of these disulphide bonds still remaining unknown, these alterations might affect the sperm’s fertilising ability [51]–[52]. In fact, protamines, the most abundant proteins in a sperm nucleus [53], condense the chromatin, protect DNA from nucleases and other damaging agents, and remove transcription factors and proteins to help reset the imprinting code in the fertilised oocyte [54]. In addition, protamines are cysteine-rich proteins that establish disulphide bonds between them. This, in turn, stabilises protein conformation [55], so that any alteration that disrupts disulfide bonds can negatively affect the protamine functions. Regarding DNA fragmentation, it is not observed immediately after thawing, but mainly after 240 min [34], and in pigs it has a much lower incidence than in other species such as human and horse [51], [56]. In the present work, levels of sperm DNA fragmentation in FT spermatozoa are higher when the HT is of 3 h than when it is of 24 h. This agrees with our data regarding the levels of free cysteine residues in sperm nucleoproteins (FT spermatozoa) that are higher when the HT is of 3 h than when it is of 24 h. Thus, it seems reasonable to hypothesise that a longer contact of sperm with seminal plasma factors during HT [2]–[3] triggers a sperm response that leads to the protection of the sperm nucleus against freeze-thawing.

Apart from the protective effects on the boar-sperm nucleus, the benefits of a longer HT in FT spermatozoa have also been observed in sperm membrane integrity (assessed by SYBR-14/PI and PNA-FITC/PI assays), permeability (assessed through YO-PRO-1/PI), and lipid disorder (assessed through M540/YO-PRO-1). Our results match with those obtained by Eriksson et al. [6], who found that post-thaw sperm viability was significantly higher when HT was longer (of 10 h or 20 h) than when it was shorter (3 h), and with those reported by Kotzias-Bandeira et al. [5], who concluded that a longer HT resulted in a higher acrosome integrity after freeze-thawing. In contrast, Guthrie and Welch [7] did not find significant differences between the same two HTs performed in this study (i.e. 3 h and 24 h) either in sperm plasma membrane integrity or in sperm motility. However, there are two aspects that we must take into account when comparing our results with those obtained by the other authors. First, Kotzias-Bandeira et al. [5], Eriksson et al. [6] and Guthrie and Welch [7] evaluated the differences between HTs within 30 min post-thawing, while in our study we have evaluated the spermatozoa not only after 30 min, but also after 240 min post-thawing, and the highest difference between both HTs has mainly been seen after 240 min rather than after 30 min. Second, all of these previous reports only evaluated plasma membrane integrity and motility of spermatozoa, whereas this study has also evaluated HT effects on the sperm nucleus, membrane permeability and lipid disorder. Finally, and even though freeze-thawing slightly increases the percentages of viable spermatozoa with high levels of H_2_O_2_ without affecting O_2_^−•^ levels, HT has no effect on ROS generated during cryopreservation. These results are not surprising in boar sperm. We must remember that, whereas with species such as horse [57], bull [58] and dog [59], sperm cryopreservation increases ROS levels, the production of ROS linked to cryopreservation is lower in pig and has a lesser impact [34], [43], [60]–[61], minimising thus the role of ROS as deleterious agents in boar-sperm cryopreservation.

As indicated above, our results indicate that the higher cryotolerance linked to a HT of 24 h is concomitant with specific changes in pSer levels of several structural sperm proteins. Specifically, mini-array analyses suggest an increase in pSer levels in four proteins, namely HSP70, GSK3, total TRK and CDK1/CDC2. These results suggest that, at least in part, seminal plasma factors and/or other factors present in extended semen confer higher cryotolerance during HT and act through serine-phosphorylation of some proteins that are involved in stress, such as HSP70. In this sense, we must remember that the presence of seminal factors in extended semen may confer higher resistance to cold shock [3]. This obviously does not preclude the existence of other mechanisms related to the phosphorylation of other residues, such as tyrosines or threonines, and/or phosphorylation/dephosphorylation of other proteins different from the 30 studied in this work. However, the putative effects of HT on pSer of GSK3, total TRK and CDC2/CDK1 are at this moment only hypothetical. This is due to two facts. The first one is that pSer changes observed in mini-arrays were of a much lower intensity than those observed in HSP70. The second fact is that we have not confirmed the specificity of the obtained results by performing further experiments involving specific immunoprecipitation and subsequent pSer analyses of the specifically immunoprecipitated GSK3, total TRK and CDC2/CDK1. For this reason, further research is warranted to better elucidate the role that the observed changes in pSer of GSK3, total TRK and CDC2/CDK1 plays on boar-sperm cryotolerance.

The interpretation of results regarding HSP70 is different from that of those from GSK3, total TRK and CDC2/CDK1. First, HSP70 presents the highest differences between both HTs. Second, our immunoprecipitation approach confirms the specificity of the anti-HSP70 antibody used in mini-array analyses, since the specific antibody immunoprecipitated a protein with the recorded molecular weight of HSP70. Furthermore, immunoprecipitation analyses have confirmed that differences between levels of phosphorylated serines observed between HTs are due to the phosphorylation of these residues for a longer HT rather than to alterations in HSP70 content. Taking this into consideration, the pSer levels observed after a HT of 24 h are half high as those observed after a HT of 3 h. Here, we must also point out that although immunoprecipitation yield/effectiveness leads that not all the samples have the same quantity of HSP70, the relevant aspect is the amount of HSP70 that is serine-phosphorylated, and this is rightly assessed through pSer-HSP70:total HSP70 ratio. Finally, it is worth noting that we have correlated sperm cryotolerance (Component 1 from PCA) with pSer-HSP70:total HSP70 ratios, and that the pSer-HSP70:total HSP70 ratio has been the only parameter included in regression analyses as being able to predict boar-sperm cryotolerance before freeze-thawing. Related to this, it is important to keep in mind that, in the case of Component 1, factor loadings (a_ij_) from cryotolerance indexes were positive in those categories including fully intact spermatozoa (i.e. %SYBR-14^+^/PI^−^ spermatozoa, %YO-PRO-1^−/^PI^−^ spermatozoa, …) but negative in those categories of non-viable/damaged spermatozoa (e.g. %SYBR-14^+^/PI^+^ spermatozoa, %YO-PRO-1^+^/PI^+^ spermatozoa, %YO-PRO-1^+^/PI^−^ spermatozoa).

The involvement of HSP70 in the modulation of cryotolerance is not surprising if we take into account that heat-shock proteins have been found in mammalian spermatozoa [17], [62]–[63]. Specifically, HSP70 is a chaperone protein involved in maintaining proper protein conformation [64]. This protein changes its distribution patterns during capacitation and acrosome reaction [65] and is involved in spermatogenesis, fertilisation and post-fertilisation events [66]–[68]. Up to now, studies relating HSP70 and sperm quality have been focused on the amounts of this protein and its correlation with sperm quality parameters, heat-stress response and thermotolerance of freshly ejaculated rather than FT spermatozoa [23], [66], [69]. However, the main finding of our work is that the involvement of this protein in boar-sperm cryotolerance seems to occur, at least in part, via signal transduction pathways that involve phosphorylation-related mechanisms, as confirmed by our immunoprecipitation studies and in a similar fashion to that observed in osmotic stress response of rhesus macaque sperm [17]. Nevertheless, the mechanism/s by which HSP70 could exert its protective role against cryopreservation is not clear. Thus, Bohring et al. [70] suggested that HSP70 is hardly been involved in a stress response because spermatozoa have highly condensed chromatin and, in this way, sperm cells are unable to develop a stress response. These authors proposed, instead, that HSP70 might mediate protein folding and the process of translocation across the sperm membrane. However, in our study we have seen that sperm samples stored for a longer HT present higher levels of serine phosphorylation than those stored for a shorter HT. This does not discard the hypothesis that HSP70 intervenes in protein folding, but it does emphasise the possibility that HSP70 also mediates stress response in sperm by activation/inactivation through serine-phosphorylation. Finally, another aspect that remains to be elucidated regards the mechanism by which a protein that, so far, has mainly been found at the equatorial region of boar spermatozoa [68] increases the sperm cryotolerance after serine phosphorylation.

To the best of our knowledge this is the first study demonstrating that a key post-translation mechanism of protein regulation such as phospho/dephosphorylation is involved in sperm cryotolerance. Indeed, previous reports have demonstrated that cryotolerance is related to the content of some proteins involved in stress response, such as HSP90AA1 [71], but no one has previously demonstrated that not only the content but also the phosphorylation status of serine residues is relevant for sperm cryotolerance, as PCA and multiple regression analyses have shown. This suggests that spermatozoa present some regulation mechanism that modulates sperm physiology during storage at 17°C and confers, among other changes, higher resistance to stressful procedures such as cold-shock and osmotic stress during sperm cryopreservation.

In conclusion, in the present report we have observed, in agreement with other studies [34], [45], [52], that freeze-thawing of boar spermatozoa impairs their plasma membrane, motility, and destabilises their nucleoprotein structure by disrupting disulphide bonds. However, the extent of this deterioration depends on the duration of the previous HT, in a concomitant manner with changes in pSer levels of some sperm proteins like HSP70. For this reason, we hypothesise that seminal plasma or other factors present in extended semen trigger a signal transduction pathway during the HT period that involves serine-phosphorylation of proteins like HSP70, and this increase, in turn, is involved in the HT-related increase of boar-sperm cryotolerance. Finally, these results open the door to further investigations centred in the study of post-translational protein modifications that may underlie sperm cryotolerance in mammalian species.

## Supporting Information

Table S1 **Effects of holding time prior to freeze-thawing on membrane integrity of boar spermatozoa, evaluated through PNA-FITC/PI assay, after 30 and 240 min post-thawing at 37°C.** Data are shown as mean ± SEM. Different superscripts (*a, b, c, d, e*) mean significant differences (*P*<0.05) among rows and columns within the same category of spermatozoa (% spermatozoa with intact plasma membrane, PNA-FITC^−/^PI^−^; % spermatozoa with damaged plasma membrane that present outer acrosome membrane, PNA-FITC^+^/PI^+^; % spermatozoa with damaged plasma membrane with lost outer acrosome membrane, PNA-FITC^−/^PI^+^; % spermatozoa with damaged plasma membrane, PNA-FITC^+^/PI^−^). (Ext: extended semen; FT: frozen-thawed spermatozoa).
(DOC)

Table S2 **Effects of holding time prior to freeze-thawing on membrane permeability (YO-PRO-1/PI assay) of boar spermatozoa after 30 and 240 min post-thawing at 37°C.** Data are shown as mean ± SEM. Different superscripts (*a, b, c, d*) mean significant differences (*P*<0.05) among rows and columns within the same category of spermatozoa (i.e. % Viable spermatozoa without changes in membrane permeability, % Viable spermatozoa with early changes in membrane permeability, Non-viable spermatozoa). (Ext: extended semen; FT: frozen-thawed spermatozoa; m.p.: membrane permeability).
(DOC)

Table S3 **Effects of holding time prior to freeze-thawing on boar sperm plasma membrane lipid disorder (M540/YO-PRO-1 assay) after 30 and 240 min post-thawing at 37°C.** Data are shown as mean ± SEM. Different superscripts (*a, b, c, d*) mean significant differences (*P*<0.05) among rows and columns within the same category of spermatozoa (i.e. % Viable spermatozoa with high membrane lipid disorder, % Viable spermatozoa with low membrane lipid disorder, Non-viable spermatozoa with high membrane lipid disorder, % Non-viable spermatozoa with low membrane lipid disorder). (Ext: extended semen; FT: frozen-thawed spermatozoa).
(DOC)

Table S4 **Effects of holding time prior to freeze-thawing on intracellular calcium levels ([Ca^2+^]_i_) (Fluo3-AM/PI assay) of boar spermatozoa after 30 and 240 min post-thawing at 37°C.** Data are shown as mean ± SEM. Different superscripts (*a, b, c, d, e*) mean significant differences (*P*<0.05) among rows and columns within the same category of spermatozoa (% Viable spermatozoa with low [Ca^2+^]_i_ (Fluo3-AM^−/^PI^−^); % Viable spermatozoa with high [Ca^2+^]_i_ (Fluo3-AM^+^/PI^−^); % Non-viable spermatozoa with low [Ca^2+]^_i_ (Fluo3-AM^−/^PI^+^); % Non-viable spermatozoa with high [Ca^2+]^_i_ (Fluo3-AM^+^/PI^+^); GMFI (FL1) Fluo3^+^ (total spermatozoa)). (Ext: extended semen; FT: frozen-thawed spermatozoa; GMFI: Geometric mean of fluorescence intensity (arbitrary units)).
(DOC)

Table S5 **Effects of holding time prior to freeze-thawing on the levels of reactive oxygen species (peroxides and superoxides) after 30 and 240 min post-thawing at 37°C.** Data are shown as mean ± SEM. Different superscripts (*a, b, c, d, e*) mean significant differences (*P*<0.05) among rows and columns within the same category of spermatozoa (i.e. % Spermatozoa DCF^+^/PI^−^; GMFI (FL1) DCF^+^/PI^−^ (Viable spermatozoa with high H_2_O_2_); GMFI (FL1) DCF^+^ (total spermatozoa); % Spermatozoa E^+^/YO-PRO-1^−^; GMFI (FL3) E^+^/YO-PRO-1^−^ (Viable spermatozoa with high O_2_^−•^; GMFI (FL3) E^+^ (total spermatozoa)). (Ext: extended semen; FT: frozen-thawed spermatozoa; GMFI: Geometric mean of fluorescence intensity (arbitrary units)).
(DOC)

Table S6 **Effects of holding time prior to freeze-thawing on the levels of reactive oxygen species (peroxides and superoxides) after 30 and 240 min post-thawing at 37°C.** Data are shown as mean ± SEM. Different superscripts (*a, b, c, d, e*) mean significant differences (*P*<0.05) among rows and columns within the same category of spermatozoa (i.e. % Spermatozoa DCF^+^/PI^−^; GMFI (FL1) DCF^+^/PI^−^ (Viable spermatozoa with high H_2_O_2_); GMFI (FL1) DCF^+^ (total spermatozoa); % Spermatozoa E^+^/YO-PRO-1^−^; GMFI (FL3) E^+^/YO-PRO-1^−^ (Viable spermatozoa with high O_2_^−•^; GMFI (FL3) E^+^ (total spermatozoa)). (Ext: extended semen; FT: frozen-thawed spermatozoa; GMFI: Geometric mean of fluorescence intensity (arbitrary units)).
(DOC)

Information S1 (DOC)

## References

[1] Rath D, Bathgate R, Rodríguez-Martínez H, Roca J, Strzezek J, et al. (2009) Recent advances in boar semen cryopreservation. Soc Reprod Fertil Suppl 66: 51–66. 19848266

[2] Tamuli MK, Watson PF (1994) Cold resistance of live boar spermatozoa during incubation after ejaculation. Vet Rec 135: 160–162. 7985346 10.1136/vr.135.7.160

[3] Johnson LA, Weitze KF, Fiser P, Maxwell WMC (2000) Storage of boar semen. Anim Reprod Sci 62: 143–172. 10924823 10.1016/s0378-4320(00)00157-3

[4] Muiño-Blanco T, Pérez-Pé R, Cebrián-Pérez JA (2008) Seminal plasma proteins and sperm resistance to stress Reprod Domest Anim. 43 Suppl 418–31. 10.1111/j.1439-0531.2008.01228.x 18803753

[5] Kotzias-Bandeira E, Waberski D, Weitze KF (1997) Cooling of boar spermatozoa prior to freezing and post-thaw quality and evaluation of membrane state using chlortetracycline (CTC) staining. Deutsche Tierärztliche Wochenschrift 104: 302–306. 9324457

[6] Eriksson BM, Vázquez JM, Martínez EA, Roca J, Lucas X, et al (2001) Effects of holding time during cooling and of type of package on plasma membrane integrity, motility and in vitro oocyte penetration ability of frozen–thawed boar spermatozoa. Theriogenology 55: 1593–1605. 11393213 10.1016/s0093-691x(01)00505-2

[7] Guthrie HD, Welch GR (2005) Impact of storage prior to cryopreservation on plasma membrane function and fertility of boar sperm. Theriogenology 63: 396–410. 15626407 10.1016/j.theriogenology.2004.09.020

[8] Urner F, Sakkas D (2003) Protein phosphorylation in mammal spermatozoa. Reproduction 125: 17–26. 12622692 10.1530/rep.0.1250017

[9] Naz RK, Rajesh PB (2004) Role of tyrosine phosphorylation in sperm capacitation/acrosome reaction. Reprod Biol Endocrinol 2: 75. 15535886 10.1186/1477-7827-2-75 PMC533862

[10] Aparicio IM, Bragado MJ, Gil MC, García-Herreros M, González-Fernández L, et al (2007) Porcine sperm motility is regulated by serine phosphorylation of the glycogen synthase kinase-3α. Reproduction 134: 435–444. 17709562 10.1530/REP-06-0388

[11] Fernández-Novell JM, Ballester J, Altirriba J, Ramió-Lluch L, Barberà A, et al (2011) Glucose and fructose as functional modulators of overall dog, but not boar sperm function. Reprod Fertil Dev 23: 468–480. 21426864 10.1071/RD10120

[12] Jha KN, Salicioni AM, Arcelay E, Chertihin O, Kumari S, et al (2006) Evidence for the involvement of proline-directed serine/threonine phosphorylation in sperm capacitation. Mol Hum Reprod 12: 781–789. 17050774 10.1093/molehr/gal085

[13] Vijayaraghavan S, Chakrabarti R, Myers K (2007) Regulation of sperm function by protein phosphatase PP1γ2. Soc Reprod Fertil Suppl 63: 111–121. 17566266

[14] Tardif S, Dubé C, Chevalier S, Bailey JL (2001) Capacitation is associated with tyrosine phosphorylation and tyrosine kinase-like activity of pig sperm proteins. Biol Reprod 65: 784–792. 11514342 10.1095/biolreprod65.3.784

[15] Travert C, Carreau S, Galeraud-Denis I (2009) Sperm capacitation: physiology. Gynecol Obstet Fertil 37: 523–528. 19477675 10.1016/j.gyobfe.2009.04.004

[16] Visconti PE (2009) Understanding the molecular basis of sperm capacitation through kinase design. PNAS 106: 667–668. 19144927 10.1073/pnas.0811895106 PMC2630107

[17] Cole JA, Meyers SA (2011) Osmotic stress stimulates phosphorylation and cellular expression of heat shock proteins in rhesus macaque sperm. J Androl 32: 402–410. 21088232 10.2164/jandrol.110.010702

[18] Naz RK, Ahmad K (1994) Molecular identities of human sperm proteins that bind human zona pellucida: nature of sperm–zona interaction, tyrosine kinase activity and involvement of FA-1. Mol Reprod Dev 39: 397–408. 7534465 10.1002/mrd.1080390408

[19] Flesch FM, Gadella BM (2000) Dynamics of the mammalian sperm plasma membrane in the process of fertilization. Biochim Biophys Acta 1469: 197–235. 11063883 10.1016/s0304-4157(00)00018-6

[20] Naz RK, Ahmad K, Kaplan P (1993) Involvement of cyclins and cdc2 serine/threonine protein kinase in human sperm cell function. Biol Reprod 48: 720–728. 8485236 10.1095/biolreprod48.4.720

[21] Paasch U, Grunewald S, Agarwal A, Glandera HJ (2004) Activation pattern of caspases in human spermatozoa. Fertil Steril 81: 802–809. 15019813 10.1016/j.fertnstert.2003.09.030

[22] Grünewald S, Paasch U, Wuendrich K, Glander HJ (2005) Sperm caspases become more activated in infertility patients than in healthy donors during cryopreservation. Arch Androl 51: 449–460. 16214731 10.1080/014850190947813

[23] Erata GO, Koçak Toker N, Durlanik O, Kadioğlu A, Aktan G, et al (2008) The role of heat shock protein 70 (Hsp 70) in male infertility: is it a line of defense against sperm DNA fragmentation? Fertil Steril 90: 322–327. 17880957 10.1016/j.fertnstert.2007.06.021

[24] Martin G Cagnon N, Sabido O, Sion B, Grizard G, et al (2007) Kinetics of occurrence of some features of apoptosis during the cryopreservation process of bovine spermatozoa. Hum Reprod 22: 380–388. 17092986 10.1093/humrep/del399

[25] Ibrahim NM, Gilbert GR, Loseth KJ, Crabo BG (2000) Correlation between clusterin-positive spermatozoa determined by flow cytometry in bull semen and fertility. J Androl 21: 887–894. 11105915

[26] Ibrahim NM, Romano JE, Troedsson MHT, Crabo BG (2001) Effect of scrotal insulation on clusterin-positive cells in ram semen and their relationship to semen quality. J Androl 22: 863–877. 11545301

[27] Breitbart H, Naor Z (1999) Protein kinases in mammalian sperm capacitation and the acrosome reaction. Rev Reprod 4: 151–159. 10521152 10.1530/ror.0.0040151

[28] Harrison RAP (2004) Rapid PKA-catalysed phosphorylation of boar sperm proteins induced by the capacitating agent bicarbonate. Mol Reprod Dev 67: 337–52. 14735495 10.1002/mrd.20028

[29] Suzuki T, Fujinoki M, Shibahara H, Suzuki M (2010) Regulation of hyperactivation by protein phosphatase 2A on hamster spermatozoa. Reproduction 139: 847–856. 20185533 10.1530/REP-08-0366

[30] De Lamirande E, Gagnon C (2002) The extracellular signal-regulated kinase (ERK) involved in human sperm function and modulate superoxide anion. Mol Hum Reprod 8: 124–135. 11818515 10.1093/molehr/8.2.124

[31] Thundathil J, de Lamirande E, Gagnon C (2002) Different signal transduction pathways are involved during human sperm capacitation induced by biological and pharmacological agents. Mol Hum Reprod 8: 811–816. 12200458 10.1093/molehr/8.9.811

[32] Aquila S, Sisci D, Gentile M, Middea E, Catalano S, et al (2004) Estrogen receptor (ER) alpha and ER beta are both expressed in human ejaculated spermatozoa: evidence of their direct interaction with phosphatidylinositol-3-OH kinase/Akt pathway. J Clin Endocrinol Metabol 89: 1443–1451. 10.1210/jc.2003-031681 15001646

[33] Jungnickel MK, Sutton KA, Wang Y, Florman HM (2007) Phosphoinositide-dependent pathways in mouse sperm are regulated by egg ZP3 and drive the acrosome reaction. Develop Biol 304: 116–126. 17258189 10.1016/j.ydbio.2006.12.023 PMC1892180

[34] Yeste M, Flores E, Estrada E, Bonet S, Rigau T, et al (2013) Reduced glutathione and procaine hydrochloride protect the nucleoprotein structure of boar spermatozoa during freeze-thawing by stabilising disulfide bonds. Reprod Fertil Dev 25: 1036–1050. 23089308 10.1071/RD12230

[35] Pursel VG, Johnson LA (1975) Freezing of boar spermatozoa: fertilizing capacity with concentrated semen and a new thawing procedure. J Anim Sci 40: 99–102. 1110222 10.2527/jas1975.40199x

[36] Casas I, Sancho S, Briz M, Pinart E, Bussalleu E, et al (2009) Freezability prediction of boar ejaculates assessed by functional sperm parameters and sperm proteins. Theriogenology 72: 930–948. 19651432 10.1016/j.theriogenology.2009.07.001

[37] Westendorf P, Richter L, Treu H (1975) Zur Tiefgefrierung von Ebersperma: Labor und Besamungsergebnisse mit dem Hülsenberger Pailletten Verfahren. Dtsch Tierärztl Wschr Deutsche Tierärztliche Wochenschrift 82: 261–267. 1104331

[38] Lee JA, Spidlen J, Boyce K, Cai J, Crosbie N, et al (2008) MIFlowCyt: The minimum information about a flow cytometry experiment. Cytometry A 73: 926–930. 18752282 10.1002/cyto.a.20623 PMC2773297

[39] Garner DL, Johnson LA (1995) Viability assessment of mammalian sperm using SYBR-14 and propidium iodide. Biol Reprod 53: 276–284. 7492679 10.1095/biolreprod53.2.276

[40] Nagy S, Jansen J, Topper EK, Gadella BM (2003) A triple-stain flow cytometric method to assess plasma- and acrosome-membrane integrity of cryopreserved bovine sperm immediately after thawing in presence of egg-yolk particles. Biol Reprod 68: 1828–1835. 12606354 10.1095/biolreprod.102.011445

[41] Harrison RAP, Ashworth PJ, Miller NG (1996) Bicarbonate/CO_2_, an effector of capacitation, induces a rapid and reversible change in the lipid architecture of boar sperm plasma membranes. Mol Reprod Dev 45: 378–391. 8916050 10.1002/(SICI)1098-2795(199611)45:3<378::AID-MRD16>3.0.CO;2-V

[42] Kadirvel G, Kumar S, Kumaresan A, Kathiravan P (2009) Capacitation status of fresh and frozen-thawed buffalo spermatozoa in relation to cholesterol level, membrane fluidity and intracellular calcium. Anim Reprod Sci 116: 244–253. 19261396 10.1016/j.anireprosci.2009.02.003

[43] Guthrie HD, Welch GR (2006) Determination of intracellular reactive oxygen species and high mitochondrial membrane potential in Percoll-treated viable boar sperm using fluorescence-activated flow cytometry. J Anim Sci 84: 2089–2100. 16864869 10.2527/jas.2005-766

[44] Petrunkina AM, Waberski D, Bollwein H, Sieme H (2010) Identifying non-sperm particles during flow cytometric physiological assessment: a simple approach. Theriogenology 73: 995–1000. 20171719 10.1016/j.theriogenology.2009.12.006

[45] Flores E, Ramió-Lluch L, Bucci D, Fernández-Novell JM, Peña A, et al (2011) Freezing-thawing induces alterations in histone H1-DNA binding and the breaking of protein-DNA disulphide bonds in boar sperm. Theriogenology 76: 1450–1464. 21855992 10.1016/j.theriogenology.2011.05.039

[46] Bradford MM (1976) A rapid and sensitive method for the quantification of microgram quantities of protein utilizing the principle of protein-dye binding. Anal Biochem 72: 248–254. 942051 10.1016/0003-2697(76)90527-3

[47] Enciso M, López-Fernández C, Fernández JL, García P, Gosálbez AI, et al (2006) A new method to analyze boar sperm DNA fragmentation under bright-field or fluoresecence microscopy. Theriogenology 65: 308–316. 15996725 10.1016/j.theriogenology.2005.05.044

[48] Fraser L, Parda A, Filipowicz K, Strzeżek J (2010) Comparison of post-thaw DNA integrity of boar spermatozoa assessed with the neutral comet assay and Sperm-Sus Halomax test kit. Reprod Domest Anim 45: 155–160. 19895391 10.1111/j.1439-0531.2009.01537.x

[49] Yeste M, Briz M, Pinart E, Sancho S, Garcia-Gil N, et al (2008) Boar spermatozoa and prostaglandin F_2α_. Quality of boar sperm after the addition of prostaglandin F2_α_ to the short-term extender over cooling time. Anim Reprod Sci 108: 180–195. 17897798 10.1016/j.anireprosci.2007.08.008

[50] Yeste M, Briz M, Pinart E, Sancho S, Bussalleu E, et al (2010) The osmotic tolerance of boar spermatozoa and its usefulness as sperm quality parameter. Anim Reprod Sci 119: 265–274. 20227204 10.1016/j.anireprosci.2010.02.011

[51] Silva PFN, Gadella BM (2006) Detection of damage in mammalian sperm cells. Theriogenology 65: 958–978. 16242762 10.1016/j.theriogenology.2005.09.010

[52] Estrada E, Rodríguez-Gil JE, Rocha LG, Balasch S, Bonet S, et al (2014) Supplementing cryopreservation media with reduced glutathione increases fertility and prolificacy of sows inseminated with frozen-thawed boar semen. Andrology 2: 88–99. 24123940 10.1111/j.2047-2927.2013.00144.x

[53] Flores E, Cifuentes D, Fernández-Novell JM, Medrano A, Bonet S, et al (2008) Freeze-thawing induces alterations in the protamine-1/DNA overall structure in boar sperm. Theriogenology 69: 1083–1094. 18359506 10.1016/j.theriogenology.2008.01.022

[54] Oliva R (2006) Protamines and male infertility. Hum Reprod Update 12: 417–435. 16581810 10.1093/humupd/dml009

[55] Linke K, Jakob U (2003) Not every disulfide lasts forever: disulfide bond formation as a redox switch. Antioxid Redox Signal 5: 425–434. 13678530 10.1089/152308603768295168

[56] Baumber J, Ball BA, Linfor JJ, Meyers SA (2003) Reactive oxygen species and cryopreservation promote DNA fragmentation in equine spermatozoa. J Androl 24: 621–628. 12826702 10.1002/j.1939-4640.2003.tb02714.x

[57] Ball BA, Vo AT, Baumber J (2001) Generation of reactive oxygen species by equine spermatozoa. Am J Vet Res 62: 508–515. 11327456 10.2460/ajvr.2001.62.508

[58] Bilodeau JF, Chatterjee S, Sirard MA, Gagnon C (2000) Levels of antioxidant defenses are decreased in bovine spermatozoa after a cycle of freezing and thawing. Mol Reprod Dev 55: 282–288. 10657047 10.1002/(SICI)1098-2795(200003)55:3<282::AID-MRD6>3.0.CO;2-7

[59] Kim SH, Yu DH, Kim YJ (2010) Effects of cryopreservation on phosphatidylserine translocation, intracellular hydrogen peroxide, and DNA integrity in canine sperm. Theriogenology 73: 282–292. 19883935 10.1016/j.theriogenology.2009.09.011

[60] Kim S, Lee YJ, Kim YJ (2011) Changes in sperm membrane and ROS following cryopreservation of liquid boar semen stored at 15°C. Anim Reprod Sci 124: 118–124. 21349666 10.1016/j.anireprosci.2011.01.014

[61] Yeste M, Estrada E, Casas I, Bonet S, Rodríguez-Gil JE (2013) Good and bad freezability boar ejaculates differ in the integrity of nucleoprotein structure after freeze-thawing but not in ROS levels. Theriogenology 79: 929–939. 23398739 10.1016/j.theriogenology.2013.01.008

[62] Naaby-Hansen S, Herr JC (2010) Heat shock proteins on the human sperm surface. J Reprod Immunol 84: 32–40. 19962198 10.1016/j.jri.2009.09.006 PMC2898571

[63] Dun MD, Aitken RJ, Nixon B (2012) The role of molecular chaperones in spermatogenesis and the post-testicular maturation of mammalian spermatozoa. Hum Reprod Update 18: 420–435. 22523110 10.1093/humupd/dms009

[64] Gething MJ, Sambrook J (1992) Protein folding in the cell. Nature 355: 33–45. 1731198 10.1038/355033a0

[65] Volpe S, Galeati G, Bernardini C, Tamanini C, Mari G, et al (2008) Comparative immunolocalization of heat shock proteins (Hsp)-60, -70, -90 in boar, stallion, dog and cat spermatozoa. Reprod Domest Anim 43: 385–392. 18226022 10.1111/j.1439-0531.2007.00918.x

[66] Huang SY, Kuo YH, Lee YP, Tsou HL, Lin EC, et al (2000) Association of heat shock protein 70 with semen quality in boars. Anim Reprod Sci 63: 231–240. 10989233 10.1016/s0378-4320(00)00175-5

[67] Matwee C, Kamaruddin M, Betts DH, Basrur PK, King WA (2001) The effects of antibodies to heat shock protein 70 in fertilization and embryo development. Mol Hum Reprod 7: 829–837. 11517289 10.1093/molehr/7.9.829

[68] Spinaci M, Volpe S, Bernardini C, De Ambrogi M, Tamanini C, et al (2005) Immunolocalization of heat shock protein 70 (Hsp 70) in boar spermatozoa and its role during fertilization. Mol Reprod Dev 72: 534–541. 16142794 10.1002/mrd.20367

[69] Vallorani C, Spinaci M, Bucci D, Tamanini C, Galeati G (2010) Effects of antioxidants on boar spermatozoa during sorting and storage Anim Reprod Sci. 122: 58–65. 10.1016/j.anireprosci.2010.07.007 20709476

[70] Bohring C, Krause E, Habermann B, Krause W (2001) Isolation and identification of sperm membrane antigens recognized by antisperm antibodies, and their possible role in immunological infertility disease. Mol Hum Reprod 7: 113–118. 11160836 10.1093/molehr/7.2.113

[71] Casas I, Sancho S, Ballester J, Briz M, Pinart E, et al (2010) The HSP90AA1 sperm content and the prediction of the boar ejaculate freezability. Theriogenology 74: 940–950. 20580074 10.1016/j.theriogenology.2010.04.021

