# Supplementary material for: The Increase in Phosphorylation Levels of Serine Residues of Protein HSP70 during Holding Time at 17°C Is Concomitant with a Higher Cryotolerance of Boar Spermatozoa
Source: PLoS One. 2014 Mar 6;9(3):e90887. doi: 10.1371/journal.pone.0090887 (PMC3946327; doi:10.1371/journal.pone.0090887)
Supplement: Table S1 — Effects of holding time prior to freeze-thawing on membrane integrity of boar spermatozoa, evaluated through PNA-FITC/PI assay, after 30 and 240 min post-thawing at 37°C. Data are shown as mean ± SEM. Different superscripts (a, b, c, d, e) mean significant differences (P<0.05) among rows and columns within the same category of spermatozoa (% spermatozoa with intact plasma membrane, PNA-FITC−/PI−; % spermatozoa with damaged plasma membrane that present outer acrosome membrane, PNA-FITC+/PI+; % spermatozoa with damaged plasma membrane with lost outer acrosome membrane, PNA-FITC−/PI+; % spermatozoa with damaged plasma membrane, PNA-FITC+/PI−). (Ext: extended semen; FT: frozen-thawed spermatozoa). (DOC) [file pone.0090887.s001.doc]

|  | ***Spermatozoa with intact plasma membrane*** | | ***Spermatozoa with damaged plasma membrane*** | | | | | |
| --- | --- | --- | --- | --- | --- | --- | --- | --- |
|  | ***PNA-FITC*^-^*/PI^-^ spermatozoa*** | | ***% PNA-FITC*^+^*/PI^+^ spermatozoa*** | | ***% PNA-FITC*^-^*/PI^+^ spermatozoa*** | | ***% PNA-FITC*^+^*/PI^-^ spermatozoa*** | |
|  | ***30 min*** | ***240 min*** | ***30 min*** | ***240 min*** | ***30 min*** | ***240 min*** | ***30 min*** | ***240 min*** |
| **Ext 3h** | 89.2 ± 4.1^a^ | 50.3 ± 2.5^b^ | 6.2 ± 0.4^a^ | 31.8 ± 1.5^b^ | 3.2 ± 0.2^a^ | 16.4 ± 0.7^b^ | 1.4 ± 0.1^a^ | 1.5 ± 0.1^a^ |
| **Ext 24h** | 87.5 ± 4.3^a^ | 47.6 ± 2.7^b^ | 7.4 ± 0.5^a^ | 35.5 ± 1.6^b^ | 3.6 ± 0.2ª | 15.2 ± 0.8^b^ | 1.5 ± 0.1^a^ | 1.7 ± 0.1^a^ |
| **FT 3h** | 49.7 ± 2.6^b^ | 25.7 ± 1.3^c^ | 46.8 ± 2.6^c^ | 68.7 ± 3.1^d^ | 1.7 ± 0.1^c^ | 3.3 ± 0.2^a^ | 1.8 ± 0.1^a^ | 2.3 ± 0.2^a^ |
| **FT 24h** | 51.9 ± 2.5^b^ | 38.1 ± 1.8^d^ | 44.2 ± 2.5^c^ | 56.4 ± 2.4^e^ | 2.3 ± 0.1^c^ | 3.8 ± 0.2^a^ | 1.6 ± 0.1^a^ | 1.7 ± 0.1^a^ |
