# Supplementary material for: The Increase in Phosphorylation Levels of Serine Residues of Protein HSP70 during Holding Time at 17°C Is Concomitant with a Higher Cryotolerance of Boar Spermatozoa
Source: PLoS One. 2014 Mar 6;9(3):e90887. doi: 10.1371/journal.pone.0090887 (PMC3946327; doi:10.1371/journal.pone.0090887)
Supplement: Table S2 — Effects of holding time prior to freeze-thawing on membrane permeability (YO-PRO-1/PI assay) of boar spermatozoa after 30 and 240 min post-thawing at 37°C. Data are shown as mean ± SEM. Different superscripts (a, b, c, d) mean significant differences (P<0.05) among rows and columns within the same category of spermatozoa (i.e. % Viable spermatozoa without changes in membrane permeability, % Viable spermatozoa with early changes in membrane permeability, Non-viable spermatozoa). (Ext: extended semen; FT: frozen-thawed spermatozoa; m.p.: membrane permeability). (DOC) [file pone.0090887.s002.doc]

|  | ***Viable spermatozoa without changes in m.p.***  ***(YO-PRO-1^-^/PI^-^)*** | | ***Viable spermatozoa with early changes in m.p***  ***(YO-PRO-1^+^/PI^-^)*** | | ***Non-viable spermatozoa***  ***(YO-PRO-1^-^/PI^+^)*** | |
| --- | --- | --- | --- | --- | --- | --- |
|  | ***30 min*** | ***240 min*** | ***30 min*** | ***240 min*** | ***30 min*** | ***240 min*** |
| **Ext 3h** | 87.9 ± 3.9^a^ | 49.2 ± 2.5^b^ | 3.3 ± 0.1^a^ | 7.1 ± 0.4^b^ | 8.8 ± 0.5^a^ | 43.8 ± 2.3^b^ |
| **Ext 24h** | 86.0 ± 3.7^a^ | 46.7 ± 2.4^b^ | 3.9 ± 0.2^a^ | 7.6 ± 0.4^b^ | 10.1 ± 0.7^a^ | 45.7 ± 2.3^b^ |
| **FT 3h** | 26.3 ± 1.6^c^ | 19.4 ± 1.3^d^ | 15.0 ± 1.0^c^ | 8.8 ± 0.6^d^ | 58.7 ± 2.9^c^ | 71.8 ± 3.3^d^ |
| **FT 24h** | 38.2 ± 1.9^e^ | 28.6 ± 1.7^c^ | 14.8 ± 0.8^c^ | 9.4 ± 0.6^d^ | 47.0 ± 2.4^b^ | 62.0 ± 3.0^c^ |
