# Supplementary material for: The Increase in Phosphorylation Levels of Serine Residues of Protein HSP70 during Holding Time at 17°C Is Concomitant with a Higher Cryotolerance of Boar Spermatozoa
Source: PLoS One. 2014 Mar 6;9(3):e90887. doi: 10.1371/journal.pone.0090887 (PMC3946327; doi:10.1371/journal.pone.0090887)
Supplement: Table S3 — Effects of holding time prior to freeze-thawing on boar sperm plasma membrane lipid disorder (M540/YO-PRO-1 assay) after 30 and 240 min post-thawing at 37°C. Data are shown as mean ± SEM. Different superscripts (a, b, c, d) mean significant differences (P<0.05) among rows and columns within the same category of spermatozoa (i.e. % Viable spermatozoa with high membrane lipid disorder, % Viable spermatozoa with low membrane lipid disorder, Non-viable spermatozoa with high membrane lipid disorder, % Non-viable spermatozoa with low membrane lipid disorder). (Ext: extended semen; FT: frozen-thawed spermatozoa). (DOC) [file pone.0090887.s003.doc]

|  | ***% Viable spermatozoa with high membrane lipid disorder***  ***(M540^+^/YO-PRO-1^-^)*** | | ***% Viable spermatozoa with low membrane lipid disorder***  ***(M540^-^/ YO-PRO-1^-^)*** | | ***% Non-viable spermatozoa with high membrane lipid disorder***  ***(M540^+^/ YO-PRO-1^+^)*** | | ***% Non-viable spermatozoa with low membrane lipid disorder***  ***(M540^-^/ YO-PRO-1^+^)*** | |
| --- | --- | --- | --- | --- | --- | --- | --- | --- |
|  | ***30 min*** | ***240 min*** | ***30 min*** | ***240 min*** | ***30 min*** | ***240 min*** | ***30 min*** | ***240 min*** |
| **Ext 3h** | 4.2 ± 0.2^a^ | 10.8 ± 0.5^b^ | 85.1 ± 4.0^a^ | 43.7 ± 2.3^b^ | 5.1 ± 0.2^a^ | 30.2 ± 1.7^b^ | 5.6 ± 0.2^a^ | 15.3 ± 0.6^b^ |
| **Ext 24h** | 4.8 ± 0.2^a^ | 10.5 ± 0.5^b^ | 83.6 ± 3.8^a^ | 42.1 ± 2.4^b^ | 5.4 ± 0.3^a^ | 31.3 ± 1.8^b^ | 6.2 ± 0.3^a^ | 16.1 ± 0.7^b^ |
| **FT 3h** | 14.6 ± 0.6^c^ | 2.2 ± 0.1^d^ | 31.5 ± 1.9^c^ | 26.7 ± 1.5^d^ | 52.7 ± 2.5^c^ | 69.5 ± 3.0^d^ | 1.2 ± 0.0^c^ | 1.6 ± 0.0^c^ |
| **FT 24h** | 7.9 ± 0.4^e^ | 4.5 ± 0.2^a^ | 45.0 ± 2.3^b^ | 34.8 ± 1.9^c^ | 45.2 ± 2.1^e^ | 58.7 ± 2.6^c^ | 1.9 ± 0.1^c^ | 2.0 ± 0.1^c^ |
