# Supplementary material for: The Increase in Phosphorylation Levels of Serine Residues of Protein HSP70 during Holding Time at 17°C Is Concomitant with a Higher Cryotolerance of Boar Spermatozoa
Source: PLoS One. 2014 Mar 6;9(3):e90887. doi: 10.1371/journal.pone.0090887 (PMC3946327; doi:10.1371/journal.pone.0090887)
Supplement: Table S4 — Effects of holding time prior to freeze-thawing on intracellular calcium levels ([Ca2+]i) (Fluo3-AM/PI assay) of boar spermatozoa after 30 and 240 min post-thawing at 37°C. Data are shown as mean ± SEM. Different superscripts (a, b, c, d, e) mean significant differences (P<0.05) among rows and columns within the same category of spermatozoa (% Viable spermatozoa with low [Ca2+]i (Fluo3-AM−/PI−); % Viable spermatozoa with high [Ca2+]i (Fluo3-AM+/PI−); % Non-viable spermatozoa with low [Ca2+] i (Fluo3-AM−/PI+); % Non-viable spermatozoa with high [Ca2+] i (Fluo3-AM+/PI+); GMFI (FL1) Fluo3+ (total spermatozoa)). (Ext: extended semen; FT: frozen-thawed spermatozoa; GMFI: Geometric mean of fluorescence intensity (arbitrary units)). (DOC) [file pone.0090887.s004.doc]

|  | ***% Viable spermatozoa with low [Ca^2+^]_i_ (Fluo3-AM*^-^*/PI^-^)*** | | ***% Viable spermatozoa with high [Ca^2+^]_i_ (Fluo3-AM*^+^*/PI^-^)*** | | ***% Non-viable spermatozoa with low [Ca^2+^]_i_ (Fluo3-AM*^-^*/PI^+^)*** | | ***% Non-viable spermatozoa with high [Ca^2+^]_i_ (Fluo3-AM*^+^*/PI^+^)*** | | ***GMFI (FL1) Fluo3^+^***  ***(total spermatozoa)*** | |
| --- | --- | --- | --- | --- | --- | --- | --- | --- | --- | --- |
|  | ***30 min*** | ***240 min*** | ***30 min*** | ***240 min*** | ***30 min*** | ***240 min*** | ***30 min*** | ***240 min*** | ***30 min*** | ***240 min*** |
| **Ext 3h** | 66.8 ± 3.1^a^ | 33.2 ± 2.0^b^ | 23.7 ± 1.5^a^ | 24.8 ± 1.6^a^ | 8.3 ± 0.9^a^ | 40.9 ± 2.2^b^ | 1.2 ± 0.1^a^ | 1.1 ± 0.0^a^ | 23.6 ± 1.4^a^ | 25.3 ± 1.6^a^ |
| **Ext 24h** | 65.0 ± 2.9^a^ | 32.1 ± 2.0^b^ | 22.4 ± 1.4^a^ | 24.1 ± 1.5^a^ | 11.3 ± 1.0^a^ | 42.6 ± 2.2^b^ | 1.3 ± 0.1^a^ | 1.2 ± 0.1^a^ | 23.1 ± 1.4^a^ | 24.8 ± 1.5^a^ |
| **FT 3h** | 26.7 ± 1.8^c^ | 14.1 ± 1.2^d^ | 15.2 ± 1.2^b^ | 13.8 ± 1.2^b^ | 57.0 ± 2.8^c^ | 70.7 ± 3.3^d^ | 1.1 ± 0.1^a^ | 1.4 ± 0.1^a^ | 13.0 ± 0.8^b^ | 17.9 ± 1.2^c^ |
| **FT 24h** | 39.2 ± 2.1^e^ | 23.3 ± 1.3^c^ | 15.3 ± 1.3^b^ | 14.5 ± 1.4^b^ | 44.5 ± 2.4^b^ | 61.0 ± 2.8^c^ | 1.0 ± 0.0^a^ | 1.2 ± 0.2^a^ | 12.4 ± 0.7^b^ | 18.5 ± 1.1^c^ |
