# Supplementary material for: The Increase in Phosphorylation Levels of Serine Residues of Protein HSP70 during Holding Time at 17°C Is Concomitant with a Higher Cryotolerance of Boar Spermatozoa
Source: PLoS One. 2014 Mar 6;9(3):e90887. doi: 10.1371/journal.pone.0090887 (PMC3946327; doi:10.1371/journal.pone.0090887)
Supplement: Table S5 — Effects of holding time prior to freeze-thawing on the levels of reactive oxygen species (peroxides and superoxides) after 30 and 240 min post-thawing at 37°C. Data are shown as mean ± SEM. Different superscripts (a, b, c, d, e) mean significant differences (P<0.05) among rows and columns within the same category of spermatozoa (i.e. % Spermatozoa DCF+/PI−; GMFI (FL1) DCF+/PI− (Viable spermatozoa with high H2O2); GMFI (FL1) DCF+ (total spermatozoa); % Spermatozoa E+/YO-PRO-1−; GMFI (FL3) E+/YO-PRO-1− (Viable spermatozoa with high O2 −•; GMFI (FL3) E+ (total spermatozoa)). (Ext: extended semen; FT: frozen-thawed spermatozoa; GMFI: Geometric mean of fluorescence intensity (arbitrary units)). (DOC) [file pone.0090887.s005.doc]

|  | ***% Spermatozoa DCF^+^/PI^-^*** | | ***GMFI (FL1) DCF^+^/PI^-^*** | | ***GMFI (FL1) DCF^+^*** | |
| --- | --- | --- | --- | --- | --- | --- |
|  | ***30 min*** | ***240 min*** | ***30 min*** | ***240 min*** | ***30 min*** | ***240 min*** |
| **Ext 3h** | 2.3 ± 0.2^a^ | 1.8 ± 0.1^b^ | 83.0 ± 4.6^a^ | 67.9 ± 3.7^b^ | 80.1 ± 4.2^a^ | 30.3 ± 1.8^b^ |
| **Ext 24h** | 2.5 ± 0.2^a^ | 1.9 ± 0.1^b^ | 84.3 ± 4.9^a^ | 68.6 ± 3.9^b^ | 83.0 ± 4.5^a^ | 32.1 ± 2.0^b^ |
| **FT 3h** | 4.0 ± 0.5^c^ | 1.2 ± 0.1^d^ | 114.9 ± 6.0^c^ | 54.2 ± 3.0^d^ | 110.3 ± 5.8^c^ | 55.4 ± 3.0^d^ |
| **FT 24h** | 3.7 ± 0.4^c^ | 1.3 ± 0.1^d^ | 105.6 ± 5.6^c^ | 50.7 ± 2.8^d^ | 102.5 ± 5.4^c^ | 51.8 ± 2.9^d^ |
