# Supplementary material for: The Increase in Phosphorylation Levels of Serine Residues of Protein HSP70 during Holding Time at 17°C Is Concomitant with a Higher Cryotolerance of Boar Spermatozoa
Source: PLoS One. 2014 Mar 6;9(3):e90887. doi: 10.1371/journal.pone.0090887 (PMC3946327; doi:10.1371/journal.pone.0090887)
Supplement: Table S6 — Effects of holding time prior to freeze-thawing on the levels of reactive oxygen species (peroxides and superoxides) after 30 and 240 min post-thawing at 37°C. Data are shown as mean ± SEM. Different superscripts (a, b, c, d, e) mean significant differences (P<0.05) among rows and columns within the same category of spermatozoa (i.e. % Spermatozoa DCF+/PI−; GMFI (FL1) DCF+/PI− (Viable spermatozoa with high H2O2); GMFI (FL1) DCF+ (total spermatozoa); % Spermatozoa E+/YO-PRO-1−; GMFI (FL3) E+/YO-PRO-1− (Viable spermatozoa with high O2 −•; GMFI (FL3) E+ (total spermatozoa)). (Ext: extended semen; FT: frozen-thawed spermatozoa; GMFI: Geometric mean of fluorescence intensity (arbitrary units)). (DOC) [file pone.0090887.s006.doc]

| ***% Spermatozoa E^+^/ YO-PRO-1^-^*** | | ***GMFI (FL3) E^+^/ YO-PRO-1^-^*** | | ***GMFI (FL3) E^+^*** | |
| --- | --- | --- | --- | --- | --- |
| ***30 min*** | ***240 min*** | ***30 min*** | ***240 min*** | ***30 min*** | ***240 min*** |
| 3.4 ± 0.3^a^ | 3.2 ± 0.3^a^ | 106.8 ± 6.2^a^ | 104.1 ± 6.0^a^ | 131.5 ± 7.1^a^ | 133.0 ± 7.2^a^ |
| 3.3 ± 0.3^a^ | 3.6 ± 0.4^a^ | 104.2 ± 5.9^a^ | 102.5 ± 6.1^a^ | 133.9 ± 7.3^a^ | 136.7 ± 7.4^a^ |
| 3.5 ± 0.4^a^ | 3.9 ± 0.4^a^ | 70.0 ± 4.0^b^ | 72.9 ± 4.1^b^ | 135.2 ± 7.0^a^ | 140.8 ± 7.5^a^ |
| 3.7 ± 0.3^a^ | 3.7 ± 0.3^a^ | 69.6 ± 3.9^b^ | 71.4 ± 4.0^b^ | 136.3 ± 7.3^a^ | 139.1 ± 7.4^a^ |
