# Supplementary material for: The Increase in Phosphorylation Levels of Serine Residues of Protein HSP70 during Holding Time at 17°C Is Concomitant with a Higher Cryotolerance of Boar Spermatozoa
Source: PLoS One. 2014 Mar 6;9(3):e90887. doi: 10.1371/journal.pone.0090887 (PMC3946327; doi:10.1371/journal.pone.0090887)
Supplement: Information S1 — (DOC) [file pone.0090887.s007.doc]

**Supplementary information for Materials and Methods**

**Flow cytometry analyses**

*General information*

Samples were evaluated through a Cell Laboratory QuantaSC™ cytometer (Beckman Coulter; Fullerton, CA, USA; Serial Number: AL300087, Technical specification at <https://www.beckmancoulter.com/wsrportal/ajax/downloadDocument/721742AD.pdf?autonomyId=TP_DOC_32032&documentName=721742AD.pdf>). This instrument was equipped with two light sources: an arch-discharge lamp and an argon ion laser (488nm) set at a power of 22mW. However, in our case, only the single-line visible light (488nm) from the argon laser was used to perform the analyses. Cell diameter/volume was directly measured with the Cell Lab Quanta™ SC cytometer employing the Coulter principle for volume assessment, which is based on measuring changes in electrical resistance produced by non-conductive particles suspended in an electrolyte solution. This system has, thus, forward scatter (FS) replaced by electronic volume (EV). Furthermore, the EV channel was calibrated using 10μm Flow-Check fluorospheres (Beckman Coulter) by positioning this size of bead at channel 200 on the volume scale.

Optical filters were also original and they were FL1, FL2 and FL3. In this system, the optical characteristics for these filters were: FL1 (green fluorescence): Dichroic/Splitter, DRLP: 550nm, BP filter: 525nm, detection width 505nm-545nm; FL2 (orange fluorescence): DRLP: 600nm, BP filter: 575nm, detection width: 560nm-590nm); and FL3 (red fluorescence): LP filter: 670nm/730nm. Signals were logarithmically amplified and photomultiplier settings were adjusted to particular staining methods. FL-1 was used to detect green fluorescence (SYBR-14, PNA-FITC, YO-PRO-1, Fluo3-AM and H_2_DFCDA), while FL3 was used to detect red fluorescence (M540, HE and PI).

Sheath flow rate was set at 4.17μl min^-1^ in all analyses, and EV and side scatter (SS) were recorded in a linear mode (in EE *vs.* SS dot plots) for a minimum of 10,000 events per replicate. The analyser threshold was adjusted on the EV channel to exclude subcellular debris (particle diameter<7 μm) and cell aggregates (particle diameter >12 μm). Therefore, the sperm-specific events were positively gated on the basis of EV and SS distributions, while the others were gated out. In some protocols, as described in Supplementary Information, compensation was used to minimise spill-over of green fluorescence into the red channel.

Information on the events was collected in List-mode Data files (.LMD). These generated files were then analysed using the Cell Lab Quanta SC MPL Analysis Software (version 1.0; Beckman Coulter) to quantify dot-plot sperm populations (FL1 *vs.* FL3) and to analyse the cytometric histograms.

Unless otherwise stated, all flurochromes used for these analyses were purchased from Molecular Probes (Eugene, Oregon, USA) and were diluted with dimethyl sulfoxide (DMSO; Sigma).

*Plasma membrane integrity (SYBR-14/PI and PNA-FITC/PI)*

Plasma membrane integrity was assessed through SYBR-14/PI and PNA-FITC/PI tests. In the first case, the LIVE/DEAD Sperm Viability Kit (Molecular Probes) was used according to the protocol described by Garner and Johnson [1]. Briefly, sperm samples were incubated at 38ºC for 10min with SYBR-14 at a final concentration of 100nM, and then with PI at a final concentration of 10µM for 5 min at the same temperature. FL-1 was used for measuring the SYBR-14 fluorescence, while PI fluorescence was detected through FL-3. After this assessment, three sperm populations were identified: i. viable green-stained spermatozoa (SYBR-14^+^/PI^-^); ii. non-viable red-stained spermatozoa (SYBR-14^-^/PI^+^), and iii. non-viable spermatozoa that were stained both green and red (SYBR-14^+^/PI^+^). Non-sperm particles (debris) were found in SYBR-14^-^/PI^-^ quadrant.

Single-stained samples were used for setting the electronic volume (EV) gain, FL-1 and FL-3 PMT-voltages and for compensation of SYBR-14 spill over into the PI channel (2.45%).

Plasma membrane integrity was also evaluated through PNA-FITC/PI. In this case, spermatozoa were stained with the lectin from *Arachis hypogaea* (peanut agglutinin, PNA) conjugated with fluorescein isothiocyanate (FITC) and with PI, according to the modified procedure described by Nagy et al. [2]. Briefly, spermatozoa were stained with PNA-FITC (final concentration: 2.5µg·mL^-1^) and PI (final concentration: 10µM) and incubated at 38ºC for 5min. PNA-FITC fluorescence was collected through FL-1 and PI fluorescence was detected through FL-3. As spermatozoa were not previously permeabilised, they were identified and placed in one of the four following populations: i. spermatozoa with intact plasma membrane (PNA-FITC^-^/PI^-^); ii. spermatozoa with damaged plasma membrane that presented an outer acrosome membrane that could not be fully intact (PNA-FITC^+^/PI^+^); iii. spermatozoa with damaged plasma membrane and lost outer acrosome membrane (PNA-FITC^-^/PI^+^); and iiii. spermatozoa with damaged plasma membrane (PNA-FITC^+^/PI^-^). In summary, after PNA-FITC/PI assay there were two main categories: a) spermatozoa with intact plasma membrane (PNA-FITC^-^/PI^-^), and b) spermatozoa that had damaged their plasma membrane and/or their outer acrosome membrane (these were represented by the other three categories: PNA-FITC^+^/PI^-^, PNA-FITC^+^/PI^+^, PNA-FITC^-^/PI^+^). Unstained and single-stained samples were used for setting the electronic volume (EV) gain, FL-1 and FL-3 PMT-voltages and for compensation of PNA-FITC spill over into the PI channel (2.45%).

*YO-PRO-1/PI assay*

Changes in the permeability of sperm plasma membrane were evaluated through co-staining with YO-PRO-1 and PI from the Membrane Permeability/Dead Cell Apoptosis Kit (Molecular Probes) with YO-PRO-1/PI for flow cytometry [3]. Samples were incubated for 5min at 38ºC with YO-PRO-1 (final concentration: 40µM) and PI (final concentration: 10µM). Green fluorescence (YO-PRO-1) was collected with FL-1 sensor, while red fluorescence (PI) was collected with FL-3 sensor. In this probe, flow-cytometry dot plots yielded four differentiated sperm populations: i. viable spermatozoa (YO-PRO-1^-^/PI^-^); ii. viable spermatozoa with early changes in membrane permeability (YO-PRO-1^+^/PI^-^); iii. non-viable spermatozoa with late changes in membrane permeability (YO-PRO-1^+^/PI^+^), and iiii. non-viable spermatozoa (YO-PRO-1^-^/PI^+^). Data from the two latter sperm populations are presented as grouped in a single category of non-viable spermatozoa (see Table S2).

Unstained and single-stained samples were used for setting the electronic volume (EV) gain, FL-1 and FL-3 PMT-voltages and for compensation of YO-PRO-1 spill over into the PI channel (5.06%).

*Membrane lipid disorder (M540/YO-PRO-1)*

Membrane lipid disorder was assessed using the co-staining protocol for Merocyanine 540 (M-540) and YO-PRO-1 described by Harrison et al. [4] and adjusted in our laboratory. Sperm samples were incubated for 10min at 38ºC with M-540 and YO-PRO-1 at a final concentration of 400µM and 40µM, respectively. The fluorescence of M-540 was detected through FL-3, while that of YO-PRO-1 was detected through FL-1. Four sperm populations were observed in flow cytometry dot plots: i. viable spermatozoa with low membrane lipid disorder (M-540^-^/YO-PRO-1^-^); ii. viable spermatozoa with high membrane lipid disorder (M-540^+^/YO-PRO-1^-^); iii. non-viable spermatozoa with low membrane lipid disorder (M-540^-^/YO-PRO-1^+^), and iiii. non-viable spermatozoa with high membrane lipid disorder (M-540^+^/YO-PRO-1^+^). Data was not compensated.

*Intracellular calcium (Fluo3-AM/PI)*

Intracellular calcium of spermatozoa was determined using the protocol described by Harrison et al. [5] and modified by Kadirvel et al. [6]. Sperm samples were incubated for 10min at 37ºC with Fluo3-AM and PI at final concentrations of 1µM and 10µM, respectively. FL-1 filter was used for collecting the fluorescence of Fluo3-AM, while PI fluorescence was detected through the FL-3 filter (FL-3). Four sperm populations were identified in dot plots: i. viable spermatozoa with low levels of intracellular calcium (PI^-^/Fluo3^-^); ii. viable spermatozoa with high levels of intracellular calcium (PI^-^/Fluo3^+^); iii. non-viable spermatozoa with low levels of intracellular calcium (PI^+^/Fluo3^-^), and iiii. non-viable spermatozoa with high levels of intracellular calcium (PI^+^/Fluo3^+^).

Unstained and single-stained samples were used for setting the electronic volume (EV) gain, FL-1 and FL-3 PMT-voltages and for compensating Fluo3-AM spill over into the PI channel (2.45%) and PI spill over into the Fluo3 channel (28.72%).

*Assessment of oxidative stress: peroxides (H_2_DCFDA/PI) superoxides and (HE/YO-PRO-1)*

ROS levels were determined through two different oxidation-sensitive fluorescent probes: 2’,7’-dichlorodihydrofluorescein diacetate (H_2_DCFDA) and hydroethidine (HE), used to analyse the intracellular content of peroxides (H_2_O_2_) and superoxide anions (O_2_^-●^), respectively [7]. Following a procedure modified from Guthrie and Welch [8], a simultaneous differentiation of viable from non-viable spermatozoa was performed, by co-staining the spermatozoa either with PI or with YO-PRO-1.

In the first case, spermatozoa were stained with H_2_DCFDA at a final concentration of 200µM and PI at a final concentration of 10µM and incubated at 25ºC for 60min in the dark. H_2_DCFDA is a stable cell-permeable non-fluorescent probe that is intracellularly de-esterified and becomes highly fluorescent 2’,7’-dichlorofluorescein (DCF) upon oxidation [8]. This DCF fluorescence was collected through FL-1, while PI fluorescence was detected through FL-3. Measurements were expressed as the geometric mean of green intensity fluorescence units (GMFI, geometric mean in FL-1) and this was used as the index of ROS generation. Unstained and single-stained samples were used for setting the electronic volume (EV) gain, FL-1 and FL-3 PMT-voltages and data was not compensated.

In the second probe, samples were stained with HE (final concentration: 4µM) and with YO-PRO-1 (final concentration: 40µM) and incubated at 25ºC for 40min in the dark [8]. Hydroethidine is freely permeable to cells and it is oxidised by O_2_^-●^ to ethidium (E) and other products [9]. Fluorescence of ethidium (E^+^) was detected through FL-3 and that of YO-PRO-1 was collected through FL-1. Data were expressed as the percentage of viable sperm with high O_2_^-●^) (high ethidium fluorescence; E^+^) and the geometric mean of red-intensity fluorescence (geometric mean channel in the FL-3). Data was not compensated.

*Correction of Data: Identification of non-DNA containing particles*

The percentage of non-DNA-containing particles (alien particles) was determined, since in some flow cytometry assessments, especially when working with cryopreserved spermatozoa, there may be an overestimation of sperm particles. According to Petrunkina and Harrison [10], alien particles such as cytoplasmic droplets, cell debris, or diluent components (as egg yolk), will often show EV/FS and SS characteristics similar to those of spermatozoa and can not thus be excluded via light scatter. For this reason, 5µL of each sperm sample coming from extended or FT spermatozoa, either at 30 or 240min after incubation at 37ºC, were diluted with 895µL of milliQ-distilled water. Samples were then stained with PI at a final concentration of 10µM and incubated at 38ºC for 3min, according to the procedure described by Petrunkina et al. [11]. Percentages of alien particles (f) were used to correct the percentages of non-stained spermatozoa (*q_1_*) in each sample and dual-staining analysis, except in SYBR-14/PI assay (i.e. YO-PRO-1/PI, PNA-FITC/PI, M540/YO-PRO-1, Fluo3-AM/PI, H_2_DFCA/PI and HE/YO-PRO-1), according to the following formula:

$$q_{1}^{'}=\frac{q_{1}-f}{100-f}\times100$$

Where *q_1_’* is the percentage of non-stained spermatozoa after correction.

**Determination of free cysteine residues in sperm nucleoproteins**

Samples were first centrifuged at 600×*g* at 17°C for 20 min and resuspended in an ice-cold 50mM Tris buffer (pH adjusted at 7.4) containing 150mM NaCl, 1% (v:v) Nonidet, 0.5% (w/v) sodium deoxycolate, 1mM benzamidine, 10µg·mL^-1^ leupeptin, 0.5mM phenylmethylsulfonyl fluoride (PMSF) and 1mM sodium orthovanadate (Na_2_VO_4_). Spermatozoa were subsequently homogenised through sonication (Ikasonic U50 sonicator, Ika Labortechnick; Staufen, Germany). Afterwards, homogenates were centrifuged at 850×*g* at 4°C for 20 min. Both the resultant supernatants and the upper layer of the pellet were discarded, and the pellets were subsequently resuspended in 500µL of PBS. The purity of this separation was determined by observation under a phase-contrast microscope (Zeiss Primo Star, Karl Zeiss; Jena, Germany) at 40× magnifications (Zeiss Plan-Achromat 40×/0.65; Karl Zeiss). Purity of samples was described as the percentage of loose heads in comparison with the presence of whole, non-fractioned sperm and separated tails in the sample. In all cases, the mean purity percentage was higher than 95% of loose heads, in comparison with other sperm presentations, such as intact sperm or cells with different types of tail rupture without separating the heads from their respective mid-pieces.

The levels of free-cysteine residues in sperm nucleoproteins were determined in the samples obtained by using the 2,2’-dithiodipyridine technique (2,2’-dipyridyl disulphide; Sigma; Saint Louis, USA) as described by Brocklehurst et al. [12]. Briefly, the 10-µL aliquots of resuspended, isolated sperm heads obtained as described above were added to 990µL of an aqueous solution of 0.4mM 2,2’-dithiodipyridine. The mixture was incubated at 37°C for 1h. Afterwards, levels of free-cysteine residues in sperm nucleoproteins were determined through spectrophotometric analysis at a wavelength of 343nm.

**DNA fragmentation analyses**

First, the lysing buffer included in the commercial kit was tempered to 22ºC and vials containing low-melting agarose were incubated at 100ºC for 5 min in a water bath. Vials were then left in another water bath at 37ºC for 5 min to equilibrate the agarose temperature. Twenty-five µL of each sperm sample (at a final concentration of 10^7^ spermatozoa·mL^-1^) were added to a vial prior to mixing it thoroughly. One drop of 25 µL containing the spermatozoa in agarose was placed onto the treated face of the slides provided with the kit and covered with a glass coverslip to avoid air-bubble formation.

Slides were placed on a cooled plate within a fridge and left at 4ºC for 5 min. The coverslip was then removed and 50µL of lysis solution per slide were added. An incubation step at 22ºC for 5 min was performed, prior to washing for 5 min with miliQ water. The slides were subsequently dehydrated by three steps of 2 min each with ethanol at 70%, 90% and 100%. Finally, sperm samples were stained with 2.5 µg·mL^-1^ propidium iodide (PI; Molecular Probes, Eugene, OR, USA) and mounted in DABCO (Sigma) antifading medium.

**Sperm motility settings**

| **Parameter** | **Range** |
| --- | --- |
| Range of particles area | 10 µm^2^–80 µm^2^ |
| Curvilinear velocity (VCL) | 1–500 µm·s^-1^ |
| Linear velocity (VSL) | 1–500 µm·s^-1^ |
| Average pathway velocity (VAP) | 1–500 µm·s^-1^. |
| Straightness coefficient (STR) | 10%–98%. |
| Linearity coefficient (LIN) | 10%–98%. |
| Wobble coefficient (WOB) | 10%–98%. |
| Amplitude of lateral head displacement (ALH) | 0 µm –100 µm. |
| Beat cross frequency (BCF) | 0 Hz–100 Hz. |

**Mini-array analysis of serine phosphorylation of 30 selected proteins**

First, sperm samples were prepared as described by Fernández-Novell et al. [13]. Briefly, samples were homogenised in 1mL of an ice-cold extraction solution made up of 15mM Tris/HCl buffer (pH at 7.5) plus 120mM NaCl, 25mM KCl, 2mM EGTA, 2mM EDTA, 0.1mM DTT, 0.5% Triton X-100, 10µg·mL^-1^ leupeptin, 0.5mM phenylmethylsulfonyl fluoride (PMSF) and 1mM Na_2_VO_4_. Next, homogenised samples were left at 4ºC for 30 min and then centrifuged at 10,000×*g* at 4ºC for 15 min. After this step, the supernatants were taken and used for evaluating the degree of serine phosphorylation of the 30 sperm proteins included in the Custom Antibody Array (Figure 1). This was performed following the standard protocol provided by the manufacturer. Briefly, protein concentration was determined through the Bradford method using a commercial kit (Quick Start™ Bradford Protein Assay; BioRad) and each sample was diluted in 2mL of extraction solution containing 1% dry milk to reach a final protein concentration of 2µg·µL^-1^. Simultaneously, the Antibody Array membranes were placed in suspension culture dishes (60mm×15mm; Corning Inc.; Corning, NY, USA) and incubated with a blocking solution made up of 150mM NaCl, 25mM Tris and 0.05% (v:v) Tween-20 (TBST; pH at 7.5), and supplemented with 5% (w/v) BSA. Blocking was performed at room temperature for 1h with slow shaking. Afterwards, membranes were co-incubated with sperm protein samples at room temperature for 2h with slow shaking. After incubation, membranes were washed three times for 15 min with TBST. Next, membranes were incubated with 10µg·mL^-1^ anti-phosphoserine horseradish peroxidase (HRP)-conjugated antibody (HM2070; Hypromatrix) diluted in TBST at room temperature for 2h with slow shaking. Samples were again washed three times, with washings of 15 min each with TBST, then incubated with peroxidase substrate (ECL-Plus Western Blotting Detection; GE Healthcare UK Ltd, Amersham, UK) and finally exposed to X-ray film (Amersham Hyperfilm ECL; GE Healthcare). The analysed proteins and their position in the mini-array are displayed in Figure 1.

**Immunoprecipitation against HSP70 and Western-blot assessments**

These samples were subsequently resuspended with 1mL of ice-cold lysing 10mM Tris-HCl buffer (pH at 7.4), containing 600mM sucrose, 10µg·mL^-1^ leupeptin, 1mM benzamidine, 1mM PMSF and 1mM Na_2_VO_4_. Then, samples were homogenised through sonication (Ikasonic U50 sonicator), and the homogenates were centrifuged at 850×*g* at 4°C for 20 min. Each sample was then added to 12.5µL of a commercial presentation of protein A-sepharose (Protein A-Sepharose 4 Fast Flow; GE Healthcare Bio-Sciences AB) which had previously been diluted in lysis buffer (made up as aforementioned) at a dilution rate of 1:1 (v:v). Afterwards, samples were incubated for 1h at 4°C in continuous shaking by utilising an orbital shaker. The same treatment was applied to 500µL of lysis buffer alone as a negative control for immunoprecipitation. Next, samples were centrifuged at 12,000×g for 10 min at 4°C and the resultant pellet was discarded. The obtained supernatants were incubated with the chosen specific antibody against HSP70 at a final dilution of 1:150. Samples were incubated with the antibody for 1h at 4°C again in continuous shaking by using an orbital shaker. Subsequently, samples were added to 30µL of a commercial presentation of protein A-sepharose (Protein A Sepharose 4 Fast Flow) previously diluted in lysis buffer at a dilution rate of 1:1 (v:v). Incubation with protein A-sepharose was maintained for 1h at 4°C in continuous shaking. After this, samples were again centrifuged at 12,000×g for 10 min at 4°C and the resultant supernatants and pellets were separated, reserving the supernatants obtained as controls for the immunoprecipitation effectiveness. The pellets obtained were resuspended in 1mL of lysis buffer and again centrifuged at 12,000×g for 10 min at 4°C. The supernatants were discarded, and the pellets were subjected to two more steps of resuspension-centrifugation, in order to completely wash the immunoprecipitates. The final pellets were resuspended in 1mL of a 50mM Tris buffer (pH adjusted to 8.0) at 4°C and a new centrifugation at 12,000×g for 10 min at 4°C was carried out. The obtained pellets that contained the immunoprecipitated proteins were finally resuspended in 20µL of a 50mM Tris/HCl buffer (pH at 7.5) added to 100mM dithiotreitol (DTT) and 1% (w/v) SDS. At this step, samples were heated at 95°C for 3 min and they were subsequently centrifuged at 12,000×g for 10min at 4°C. The obtained supernatants that contained the immunoprecipitated proteins were stored at -80°C until Western blot analyses were performed.

Western blot was carried out in samples subjected to SDS gel electrophoresis, followed by transferring the electrophoreted samples to nitrocellulose membranes. With this purpose, immunoprecipitated samples were incubated with 2X loading buffer (containing 0.5% bromophenol blue) for 5 min at 90ºC, and loaded into 1.5mm SDS-gels containing 10% (w/v) acrylamide in separating gel and 4% acrylamide in stacking gel. Following the manufacturer’s instructions of the immunoprecipitation kit, 15µl per sample were loaded in each lane and electrophoresis was performed at 170V for 60 min. Next, proteins from the gels were transferred to nitrocellulose membranes for 45 min at 100mA. Membranes were then rinsed for 5 min with washing solution (Tris-buffered saline (TBS) containing 0.1% (v:v) Tween20) at room temperature and in agitation, prior to incubation with blocking solution (TBS with 5% (w/v) BSA and 0.1% (v:v) Tween 20) at 4ºC for 1h in agitation. The transferred samples were tested either with the mouse anti-HSP70 antibody (ABI-SPA-810) or with a rabbit anti-phosporylated serines antibody (Rabbit polyclonal IgG, Hypromatrix HM2070). In both cases the dilution factor (v:v) was of 1:1000. Immunoreactive proteins were detected by using HRP-conjugated goat anti-mouse (Polyclonal Rabbit Anti-Mouse immmunoglobulins/HRP, Ref: P0260-DAKO) or anti-rabbit secondary antibody (Santa Cruz Technologies; Santa Cruz, CA, USA), respectively, and the reaction was developed with an ECL-Plus detection system (Amersham; Buckinghamshire, UK). The intensity of the marks obtained was quantified using specific software for image analysis of blots (Multi Gauge v3.0; Fujifilm Europe; Düsseldorf, Germany), in which the background was previously standardised for all of the samples analysed.

**References cited in Supplementary Information for Materials and Methods**

1. Garner DL, Johnson LA (1995) Viability assessment of mammalian sperm using SYBR-14 and propidium iodide. Biol Reprod 53: 276-284.

2. Nagy S, Jansen J, Topper EK, Gadella BM (2003) A triple-stain flow cytometric method to assess plasma- and acrosome-membrane integrity of cryopreserved bovine sperm immediately after thawing in presence of egg-yolk particles. Biol Reprod 68: 1828-1835.

3. Martin G, Sabido O, Durand P, Levy R (2004) Cryopreservation induces an apoptosis-like mechanism in bull sperm. Biol Reprod 71: 28-37.

4. Harrison RAP, Ashworth PJ, Miller NG (1996) Bicarbonate/CO_2_, an effector of capacitation, induces a rapid and reversible change in the lipid architecture of boar sperm plasma membranes. Mol Reprod Dev 45: 378-391.

5. Harrison RAP, Mairet B, Miller NGA (1993) Flow cytometric studies of bicarbonate mediated Ca2^+^ influx in boar sperm populations. Mol Reprod Dev 35: 197-208.

6. Kadirvel G, Kumar S, Kumaresan A, Kathiravan P (2009) Capacitation status of fresh and frozen-thawed buffalo spermatozoa in relation to cholesterol level, membrane fluidity and intracellular calcium. Anim Reprod Sci 116: 244-253.

7. Murillo MM, Carmona-Cuenca I, Del Castillo G, Ortiz C, Roncero C, et al. (2007) Activation of NADPH oxidase by transforming growth factor-beta in hepatocytes mediates up-regulation of epidermal growth factor receptor ligands through a nuclear factor-kappaB-dependent mechanism. Biochem J 405: 251-259.

8. Guthrie HD, Welch GR (2006) Determination of intracellular reactive oxygen species and high mitochondrial membrane potential in Percoll-treated viable boar sperm using fluorescence-activated flow cytometry. J Anim Sci 84: 2089-2100.

9. Zhao H, Kalivendi S, Zhang H, Joseph J, Nithipatikom K, et al. (2003) Superoxide reacts with hydroethidine but forms a fluorescent product that is distinctly different from ethidium: potential implications in intracellular fluorescence detection of superoxide. Free Radical Biol Med 34: 1359-1368.

10. Petrunkina AM, Harrison RAP (2010) Systematic misestimation of cell subpopulations by flow cytometry: a mathematical analysis. Theriogenology 73: 839-847.

11. Petrunkina AM, Waberski D, Bollwein H, Sieme H (2010) Identifying non-sperm particles during flow cytometric physiological assessment: a simple approach. Theriogenology 73: 995-1000.

12. Brocklehurst K, Stuchbury T, Malthouse JP (1979) Reactivities of neutral and cationic forms of 2,2-dipyridyl disulphide towards thiolate anions. Detection of differences between the active centres of actinidin, papain and ficin by a three-protonic-state reactivity probe. Biochem J 183: 233-238.

13. Fernández-Novell JM, Ballester J, Altirriba J, Ramió-Lluch L, Barberà A, et al. (2011) Glucose and fructose as functional modulators of overall dog, but not boar sperm function. Reprod Fertil Dev 23: 468-480.
